# Statistical analyses plan for “MAGnItude of cigarette substitutioN after initiation oF e-cigarettes and its ImpaCt on biomArkers of exposure and potenTial harm in dual users”: MAGNIFICAT trial^☆^

**DOI:** 10.1016/j.heliyon.2024.e39695

**Published:** 2024-10-22

**Authors:** Jonathan Belsey, Jakub Weglarz, Max Scherer, Nikola Pluym, Riccardo Polosa

**Affiliations:** a JB Medical, London, UK; b ECLAT Srl, Spin-off of the University of Catania, Italy; c ABF Analytisch-Biologisches Forschungslabor GmbH, Planegg, Germany; d Department of Clinical and Experimental Medicine (CLINMED), University of Catania, Italy; e Centre for the Prevention and Treatment of Tobacco Addiction, University Teaching Hospital "Policlinico-S.Marco", Catania, Italy; f CoEHAR, University of Catania, Italy

**Keywords:** *Smoking cessation*, *Biomarkers*, *Dual use smokers*, *Smoking reduction*, *Combustion-free nicotine alternatives*

## Abstract

**Background:**

The substitution of combustible cigarettes (CC) with non-combustible nicotine alternatives, such as e-cigarettes (EC), significantly lowers exposure to harmful chemicals. However, many individuals who use ECs continue smoking CCs, becoming dual users and remaining at increased risk of toxin exposure. This study will examine how the reduction in cigarettes smoked per day (CPD) after switching to ECs correlates with changes in biomarkers of exposure (BoE) and potential harm (BoPH) to evaluate the extent of harm in dual users.

**Methods:**

The study is a prospective, non-randomised, observational, longitudinal cohort study in which 250 CC smokers will be invited to use EC as part of a smoking reduction/cessation strategy. Participants will be grouped in five dual use categories based on the change from baseline in CPD at 1, 3 and 6 months, based on self-reported use in the preceding 30 days. The primary outcome of interest is the mean absolute urinary 2-cyanoethyl mercapturic acid (2CyEMA), a well-validated BoE, also assessed at 1, 3 and 6 months. Other BoE will be assessed, together with exploration of the association between varying intensities of dual use and BoPH. The association between the dual use category and levels of BoE will be evaluated using analysis of variance (ANOVA), in order to understand which BoE are best suited to identifying different levels of dual use. Exploratory assessment of the value of BoPH will similarly be evaluated, to assess whether their predictive power extends from the binary smoker/non-smoker categorization to a more nuanced dual use categorization. The specific details of the own-brand CC will be available once smokers' enrollment in the study is complete. The EC used in the study will be the KIWI 2 Pen refillable pod system, with participants given the option to choose from three different flavored e-liquids: LEAF, MIDWAY, and GLACIAL, all containing 0.9 % nicotine. For specialized equipment used for biomarker analysis please refer our previous publication: https://pubmed.ncbi.nlm.nih.gov/38584934/

**Conclusion:**

EC are increasingly adopted as a harm reduction strategy in CC smokers who wish to quit smoking, the outlined **Statistical Analysis Plan (SAP)** provides a robust approach to understand the impact of EC on CC use and to quantify the impact of dual use on clinical outcomes using BoPH. For statistical calculations we will use R software.

## Abbreviations

AE: Adverse event
ANOVA: Analysis of Variance
BoE: Biomarkers of exposure
BoPH: Biomarkers of potential harm
CC: Combustible cigarettes
CeVal: Cyanoethylvaline
CPD: Cigarettes smoked per day
CRF: Case Report Form
2CyEMA: 2-Cyanoethyl mercapturic acid
EC: e-cigarettes
eCO: exhaled CO
NNAL: 4-(methylnitrosamino)-1-(3-pyridyl)-1-butanol
PG: Propylene glycol
SAP: Statistical Analysis Plan

## 1 Introduction

### 1.1 Preface

The scope of this SAP (Statistical Analyses Plan) paper is to outline the statistical methods and processes for analyzing data from the ongoing clinical trial. It covers the aims, study design, methods for data collection, statistical analysis approaches, sample size determination, procedures for managing data, and strategies for presenting the findings.

The full details of the protocol have previously been published – the current paper expands and develops the statistical methods outlined in the main protocol [1]. A brief summary of the objectives and methods of proposed study is described below.

The use of combustion-free nicotine alternatives, such as ECs, as a substitute for CCs has been shown to significantly reduce exposure to toxic chemicals compared to CC use [[2], [3], [4], [5]]. In this study, participants will select the ECs of their own liking, and they will be instructed to use them as much as they like. This method has been increasingly adopted as a harm reduction strategy by smokers seeking to quit [[6], [7], [8], [9]].

However, a significant proportion of those using EC continue to smoke CC (dual users) [[10], [11], [12], [13], [14]]. In these individuals, the extent to which the harmful consequences of CC use are mitigated is not clearly defined in the literature [[15], [16], [17], [18], [19]]. The uncertain benefit of dual use potentially reflects wide heterogeneity in the relative proportions of CC and EC use in dual users [20]. Further potential confounders include the unverified use of self-reporting in many of the available studies, with only limited use of quantitative biomarkers of exposure (BoE) as a validation tool and a relatively short duration of follow-up [[21], [22], [23]].

We aim to address these shortcomings by exploring the significance of a broad spectrum of BoE in the profiling of dual users based on the variation in the number of cigarettes smoked per day (CPD), with our analysis spanning follow-up periods of 1 month, 3 months and 6 months. Additionally, we will investigate the relationship between different intensities of dual use and biomarkers of potential harm (BoPH).

We will undertake a thorough assessment of the reduction in CC consumption, thus classifying dual users into distinct categories based on the extent of decrease in CC consumption. This categorization will adopt a multi-faceted approach, incorporating consumption logs, and usage of tracking application.

This report details the statistical methods and reasoning used to analyze both BoEs and BoPHs in a six-month quasi-experimental study involving 250 smokers. These participants were instructed to switch from their usual tobacco cigarette brand to a prefilled POD e-cigarette device.

These analyses will assess.

• The correlation between the extent of dual use of CC/EC and measured absolute levels of a single well-validated BoE: Urinary 2-cyanoethyl mercapturic acid (2CyEMA).
• A secondary comparison of a range of different urinary and plasma BoE targets to assess the ability of each to distinguish between varying levels of dual use.
• Exploratory assessment of:
  o An extended range of BoE.
  o The extent to which sustained dual use of CC/EC has the capacity to influence BoPH.

The study was approved by the Bioethics Committee of the District Medical Chamber in Warsaw, Poland, Approval Number: No. 04/24 of February 22, 2024.

## 2 Study objectives and endpoints

### 2.1 Study objectives

• The primary objective of the study is to characterize dual use at 1, 3 and 6 months, according to the percentage change from baseline in self-reported CPD in the preceding 30 days, and to assess the extent to which measurements of urinary 2CyEMA are able to correctly identify the dual use category.
• The secondary objective is to repeat the primary analysis using three further well documented BoE, in order to identify which of the available biomarkers has the best potential to serve as an indicator of dual use.
• Further exploratory objectives will screen a wider range of BoE candidates and additionally assess the correlation between dual use category, assessed by long term (6 month) self-reported CC/EC usage, and the risk of adverse health outcomes, assessed by a range of BoPH.

### 2.2 Endpoints

#### 2.2.1 Dual use categorization (assessed at 1 month, 3 months and 6 months from baseline)

At each analysis point, participants will be categorized according to change from baseline in CPD use, based on daily diary documentation for the 30 days preceding the assessment point. Five dual-use categories will be defined.

a) “Smoker”: No reduction or increase in CC consumption compared to baseline
b) “Poor dual use”: a 1 %–49 % reduction in CPD from baseline
c) “Moderate dual use”: a 50 %–79 % reduction in CPD from baseline
d) “Intensive dual use”: a 80 %–99 % reduction in CPD from baseline
e) “Quitter”: a complete 100 % reduction in CPD from baseline

Individuals can migrate from one category to another over the course of the study, with each timepoint being regarded as an independent analysis.

#### 2.2.2 Primary endpoint (assessed at 1 month, 3 months & 6 months from baseline)

A. Urinary 2CyEMA, normalized for urinary creatinine (μg/g creatinine)

#### 2.2.3 Secondary endpoints (assessed at 1 month, 3 months & 6 months from baseline)

Three additional candidate BoE.

a. Whole blood CeVal (Cyanoethylvaline, pmol/g globin)
b. Urinary NNAL (4-(methylnitrosamino)-1-(3-pyridyl)-1-butanol, ng/g creatinine)
c. Urinary PG (Propylene glycol, mg/g creatinine)

#### 2.2.4 Exploratory endpoints (BoE)

Additional candidate urinary BoE, potentially including, but not limited to.

a. 1-Naphthol
b. 1-Hydroxyphenanthrene
c. 1-Hydroxypyrene
d. 2-aminonaphthalene
e. 2-Hydroxypropylmercapturic acid
f. 2-Naphthol
g. 2-Hydroxyphenanthrene
h. 3-Aminobiphenyl
i. 3-Hydroxypropylmercapturic acid
j. 3-Hydroxybenzo[a]pyrene
k. 3-Hydroxyphenanthrene
l. 4-Aminobiphenyl
m. 4-Hydroxyphenanthrene
n. 9-Hydroxyphenanthrene
o. Acrylamidemercapturic acid
p. Benzo[a]pyrene-5,6,7,8-tetrahydrotetrol
q. Dihydroxypropylmercapturic acid
r. Glycidamidemercapturic acid
s. Hyrdoxyethylmercapturic acid
t. Hydroxypropylmethylmercapturic acid
u. Isopropylmercapturic acid
v. Monohydroxybutylmercapturic acid
w. N-Nitrosonornicotine
x. ortho-toluidine
y. Phenylethylmercapturic acid
z. S-Benzylmercapturic acid
aa. S-Phenylmercapturic acid
bb. Total Nicotine, Cotinine, trans-3′-Hydroxycotinine

#### 2.2.5 Exploratory endpoints (BoPH)

A. Long-term change in reported use:
  a. Participants categorized as for the primary outcome, limited to those individuals categorized in the same single dual use group at all three primary outcome assessment points (1 month, 3 months, 6 months).
  b. Participants categorized as for the primary outcome but based on mean CPD over the entire 6-month follow-up period.
  B. Candidate BoPH, including, but not limited to:
a. Biochemical markers
  i. 2,3-dinor-Thromboxane B2
  ii. 11-dehydro-Thromboxane B2
  iii. 8-iso-Prostaglandin F2alpha
  iv. Leukotriene E4
  v. soluble intercellular adhesion molecule-1
  vi. Growth differentiation factor 15
b. Physiological markers
  i. VO2max (by Chester step test)
  ii. Respiratory Symptom Evaluation Score (RSES)
  iii. Spirometric parameters including, but not limited to FEV1, FVC, FEV1/FVC, FEF25-75 + FEV 5

## 3 Study methods

### 3.1 General study design and plan

The study is a prospective, non-randomised, observational, longitudinal cohort study, with descriptive and exploratory outcomes.

Participants are invited to receive EC as part of a smoking reduction/cessation strategy and will be assigned, post hoc, to an analytical category based on the extent to which they have been able to reduce CC consumption. The specific details of the own-brand CC will be available once smokers' enrollment in the study is complete. The EC used in the study will be the KIWI 2 refillable pod system, with participants given the option to choose from three different flavored e-liquids: LEAF, MIDWAY, and GLACIAL, all containing 0.9 % nicotine. For specialized equipment used for biomarker analysis please refer to the protocol paper:https://pubmed.ncbi.nlm.nih.gov/38584934/

The primary purpose of the study is to evaluate the extent to which objective BoE are able to distinguish between varying levels of dual use of CC/EC, as documented by self-report.

Participants will be assessed at baseline, 1 month, 3 months and 6 months following recruitment. Participants will be asked to self-document both EC and CC consumption using a mobile phone application, which will ask the same four questions each day.

a) Did you smoke CC yesterday?
b) If yes, how many?
c) Did you use EC yesterday?
d) If yes, how many sessions ?

Our dedicated mobile application is asking daily 4 questions (Fig. 1), like presented on the picture below. Aim of its implementation is to gather pool of data, allowing to quantify the changes in model of use among smokers, who are becoming dual-users and be able to calculate the level of substitution as well as allocate these variables within the timeline.

**Picture 1:**
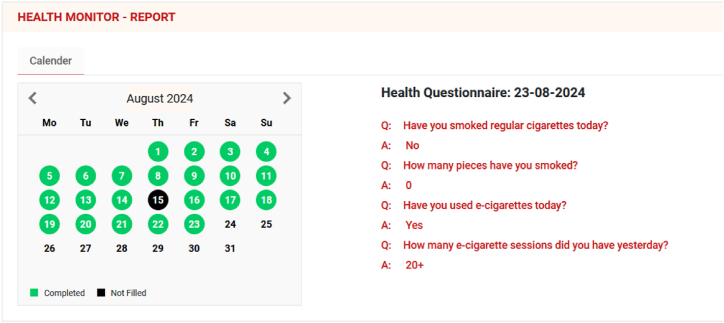
Admin view of data collection application (eDiary tool), showcasing questions asked daily and some answers; calendar on left presents frequency of answers provided by Participant.

Collected data will be complementary to the results of medical procedures, conducted during V1-V4 (between Visit 1 to Visit 4) on each of Participants, showcasing wide range of medical and behavioral aspects connected to the topic of the trial. It will also allow us to control the scale of substitution between ECs and CCs. Statistical analyses will be carried out in R v4.4.1 (R Core Team http://www.R-project.org/).

### 3.2 Inclusion-Exclusion Criteria and general study population

Adult smokers will be initially recruited from the clinical study site's participant pool and through public advertisements. Before enrollment, each individual will be offered access to a smoking cessation program designed to assist in quitting. Only those who decline participation in this cessation program will be eligible for recruitment into the study.

The study aims to recruit 250 smokers who are interested in either quitting or reducing their consumption of combustible cigarettes (CCs) by transitioning to an e-cigarette (EC) product.

Participants must meet the following criteria during both the screening (day −14) and Visit 1 (day 0):

**Inclusion Criteria**.

• Age 19 or older
• Current smokers of CCs (smoking ≥15 cigarettes per day)
• A history of regular smoking for at least 12 continuous months
• Verified smoking status (exhaled carbon monoxide [eCO] ≥ 7 ppm)
• Declined participation in a smoking cessation program
• Physically and mentally healthy, as determined by the principal investigator through medical history, vital signs (blood pressure, pulse), and spirometry
• Provided written informed consent to participate in the study

**Exclusion Criteria**.

• A prior intention to quit smoking within the next 30 days
• Any clinically significant cardiovascular, respiratory, psychiatric, or other major conditions that, in the opinion of the principal investigator, could compromise participant safety or the validity of the study outcomes
• Regular use of medications that may affect the cyclooxygenase pathway or are known to strongly induce or inhibit CYP450 enzymes within 14 days of screening or during the study; the use of hormonal contraceptives and over-the-counter pain medications is permitted
• A significant history of alcohol or drug abuse, as assessed by the principal investigator
• Use of any nicotine or tobacco-containing products other than CCs within 3 months prior to screening
• Use of nicotine replacement therapy or other smoking cessation treatments within 3 months prior to screening
• Pregnancy, breastfeeding, or planning to become pregnant during the study period
• Current participation in another clinical trial

### 3.3 Randomization and blinding

No randomization or blinding will be implemented.(see Table 1).

**Table 1:** List of procedures which will be conducted on each of Participants within particular visit (V1 – V4).

Procedure	Screening visit	Visit 1	Visit 2	Visit 3	Visit 4
	Day −14 to Day −1	Day 0	Day 28 ±3 days	Day 91 ±7 days	Day 182 ±7 days
Informed consent	X				
Inclusion/exclusion criteria	X	X			
Demographics^1^	X				
Medical/Surgical History	X				
Smoking History	X				
Cigarette consumption	X	X	X	X	X
Exhaled carbon monoxide	X	X	X	X	X
Questionnaire FTCD		X			
Weight, height (only at 1st visit), and BMI		X	X	X	X
Blood pressure, pulse rate	X	X	X	X	X
Interest to try ECs (Y/N)^2^	X				
EC familiarization		X			
EC use checks			X	X	X
Tracker APP (eDiary) Installation		X			
Tracker App (eDiary) Training		X			
Lung function/spirometry	X		X	X	X
Chester Step test (VO_2_max)		X	X	X	X
Questionnaire: RSES, MTSS, PRI-P, BIIC		X	X	X	X
eDiary verfication/eCRF		X	X	X	X
Provision of urine collection kit	X	X	X	X	
Urine pregnancy test	X	X	X	X	X
Collection of biospecimens urine and blood		X	X	X	X
Collection of exhaled breath				X	
Adverse events reporting/e-CRF		X	X	X	X
Provision of vaping products (dual-user)		X^3^	X^3^	X^3^	

The project timeline includes a screening and baseline visit (Visit 1), followed by three subsequent clinical appointments at 28 days (Visit 2), 91 days (Visit 3), and 182 days (Visit 4), as outlined in the table above.

### 3.4 Study assessments

.

## 4 Sample size

The study has a prospective cohort design, with the objective to characterize the predictive potential of various BoE as markers of dual CC/EC use in a population of smokers with varying degrees of CC substitution. There are currently no published data of this kind in the literature, so this study should be considered primarily exploratory.

Estimated sample size was based on the primary outcome. The null hypothesis to be evaluated is that the mean urinary 2CyEMA levels across the different dual-use categories originate from the same population. This hypothesis will be tested separately at 1, 3, and 6 months, with dual-use categories being reassigned at each time point based on participants' EC use in the prior 30 days, if needed.

In the absence of prior published data for this outcome and based on a fixed-effects analysis of variance (ANOVA) analysis, it was assumed that a medium effect size would be observed (f = 0.25) between the five groups under analysis. Based on α = 0.05 and 1-β = 0.8, we estimated a required total sample size of 200, or 40 participants per group. (Calculated using G∗Power v3.1.9.7).

## 5 General analysis considerations

### 5.1 Timing of analyses

The final analysis will be conducted once all participants have either completed the 6-month follow-up visit or withdrawn from the study.

### 5.2 Analysis populations

#### 5.2.1 Full analysis (FA) population

• Includes all participants who received an EC, completed at least one post-baseline assessment, and reported smoking CCs at least once in the 30 days prior.
• The FA population will be used for the primary, secondary, and exploratory BoE analyses.

#### 5.2.2 Per protocol (PP) population

• Includes participants from the FA population who completed assessments at 1, 3, and 6 months, with no major protocol deviations.
• The PP population will be used for exploratory BoPH analyses.

#### 5.2.3 Safety population

• Includes all participants who received any study treatment, excluding those who withdrew before receiving treatment.
• This population will be used for safety analysis.

### 5.3 Missing data

Individual endpoint analyses will be carried out based only on participants with complete data at the relevant time-point. Participants who have withdrawn from the study or who have been lost to follow-up will be excluded from analysis at any time point beyond their last recorded data collection.

Participants that continue in the trial but for whom there are missing BoE data points at the 1-month or 3-month assessment or missing BoE/BoPH data points at the 6-month assessment will be excluded from primary analyses related to the specific absent parameters. The participant will not be excluded from other time points and/or endpoint assessments for which full data is available.

Our experience from other studies is that missing biomarker data is rare and reflects either a failure of specimen transport or technical failure in the analytical equipment. It would therefore considered to be data “missing completely at random”. In this circumstance, and given that the basis of this study is a longitudinal repeat-measures analysis at individual subject level, imputation of missing results based on marker trends seen in other subjects in the same dual use category is unlikely to be meaningful. We consequently propose to carry out a scenario analysis using a last reading carried forward approach (ie no change in the biomarker over the time period of interest) in order to explore the impact of missing data.

Missing CPD diary data is permitted up to a maximum of 5 days within the 30-day period. Within this 5-day window, imputation of missing data points will not be undertaken – the percentage CPD for the period will be calculated based on the available data points. Where more than five diary entries are missing for any given evaluation period, the primary approach will be to exclude the participant's data from the analysis population for that analysis period only but will not be excluded from other time points for which full diary data is available.

As discussed above, the unit of analysis in this study is the individual patient, with documented CPD use over any given time window being used to determine the dual-use category for that analysis period. In this context, conventional multiple imputation methods is unlikely to be a helpful approach. We therefore propose to undertake a simple scenario analysis, based on the assumption that subjects with missing data have the same CPD consumption levels as was observed in the preceding qualifying time period.

## 6 Summary of study data

Continuous variables will be presented with descriptive statistics, including the sample size (excluding missing data), mean, standard deviation, median, and interquartile range. For categorical variables, the number and percentage of observed values (calculated from the non-missing data) will be provided. Summary tables will feature separate columns for each dual-use category, with annotations showing the total population size pertinent to the table or treatment, as well as any missing data points.

### 6.1 Subject disposition

The following variables derived from the CRF (Case Report Form) will be used to determine subject deposition.

• Screened population
• Attended visit 1 (baseline)
• Attended visit 2 (1 month)
• Dual use category membership at visit 2
• Attended visit 3 (3 months)
• Dual use category membership at visit 3
• Attended visit 4 (6 months)
• Dual use category membership at visit 4
• Study withdrawals (time + reason)
• Lost to follow-up (time)
• Deaths (subset of withdrawals)

Summary statistics will be produced in accordance with the specification in section 9.

### 6.2 Derived variables

At each evaluation point (1 month, 3 months, and 6 months), the number of cigarettes smoked per day (CPD) over the preceding 30 days will be gathered from the case report forms (CRF). The mean CPD during this period will be calculated, and the percentage reduction from baseline will be determined. This percentage will then be used to categorize participants into the following dual-use categories.

• **"Smoker"**: No change or increase in CC use from baseline.
• **"Poor dual use"**: A reduction in CPD of 1%–49 % from baseline.
• **"Moderate dual use"**: A reduction in CPD of 50%–79 % from baseline.
• **"Intensive dual use"**: A reduction in CPD of 80%–99 % from baseline.
• **"Quitter"**: A 100 % reduction in CPD from baseline.

### 6.3 Protocol deviations

Major deviations from the protocol will be defined by one or more of the following conditions.

• Failure to attend one or more assessment visits within the specified timeframes (see Section 5.4).
• More than five missing entries in the CPD diary during the 30 days prior to an assessment.
• Any missing biological samples (blood, urine) at any assessment visit.
• Use of nicotine replacement therapies or non-study EC products.

Deviations from the protocol will be documented for each evaluation point individually.

### 6.4 Demographic and baseline variables

The following variables will be identified and recorded at the screening or baseline visit.

• Age
• Sex
• Ethnicity
• Duration of smoking
• Past cessation periods (if > 6 months)
• Current CPD
• Prior use of EC (define type and duration)
• Significant past medical history
• Current prescribed medications
• Body mass index
• Blood pressure
• Ankle brachial index (ABI)
• VO2 max/Chester step test
• Respiratory Symptoms Questionnaire (RSQ)
• Baseline values for all BoE and BoPH, as defined in paragraph 5.2

Tables will be presented for the total population and broken down by the dual use category at each analysis point. Summary statistics will be produced in accordance with the specification in section 9. No statistical tests of between-groups differences will be undertaken but descriptive analyses of the differences both between groups and within groups over time will be presented.

### 6.5 Concurrent illnesses and medical conditions

Concurrent illnesses active at the time of study recruitment will be documented using ICD-10 codes.

## 7 Analyses

All values for BoE and BoPH variables at each time point will be listed by subject. Data will be summarized by dual use category. N, mean, standard deviation, median and interquartile range will be used to summarize the effect variables.

All analyses of the effect variables (BoE, BoPH) will be performed as analysis of variance according to dual use category. Differences will be tested at the 5 % significance level. The Benjamini-Hochberg false discovery procedure will be used to control for across-markers multiple comparison.

### 7.1 Primary analysis

The primary analysis will be carried out on the FA populations. The null hypothesis to be tested is that the mean absolute values for urinary 2CyEMA for each of the dual use categories is derived from the same population. The hypothesis will be tested independently at 1 month, 3 months, and 6 months, with the dual use categories being re-assigned, where necessary, at each time point.

It has been proven that a robust biomarker is ideally specific to the use of one nicotine/tobacco product. For this study, we plan to recruit sole CC smokers who are encouraged to substitute their CCs with ECs during the study. It is well-reported that the levels of biomarkers of exposure in dual users are primarily driven by smoking. Therefore, our rationale was to select a specific combustion marker. Exposure to the combustion product acrylonitrile is highly specific to smoking. The urinary metabolite 2-cyanoethylmercapturic acid (2CyEMA) was recently suggested to distinguish smoking from other forms of nicotine and tobacco use with a very strong dose response [[24], [25], [26], [27]]. Hence, in the current set-up, the quantification of 2CyEMA in dual users may allow for verification of the self-reported magnitude of CC substitution with EC usage.

Analysis will be carried out using one-way ANOVA at each time-point. As the dual use groups will be re-defined for each analysis, based on EC use in the preceding 30 days, each analysis will be treated as independent, rather than a repeated measure.

Previous publications using BoE suggest that there is a high likelihood that the biomarker data will be non-normally distributed and there is no guarantee that there will be equal variance between the categories. To address this potential issue, sandwich variance estimators will be used rather than ordinary least squares in the ANOVA regression [28,29].

In the event that the null hypothesis is rejected, pairwise comparisons will subsequently be carried out using the approach of Conover [30].

### 7.2 Secondary analyses

#### 7.2.1 Analyses of secondary endpoints

The same analytical approach as described for the primary analysis (section 7.1) will be applied to each of the three secondary BoE endpoints: whole blood CeVal, urinary NNAL and urinary PG, at 1 month, 3 months and 6 months. The analyses will be carried out using the FA population for the intervention group.

### 7.3 Exploratory efficacy analyses

#### 7.3.1 Analyses of alternative BoE

Results for a wide range of other potential BoE, listed in section 2.2, will be pre-screened for their potential to serve as a useful metric in dual users. The specific BoE will be analyzed using the same analytical approach as outlined in section 7.1.

#### 7.3.2 Analyses of BoPH

For these analyses, the dual use categories will be assessed based on long term CC usage. The statistical analysis will be carried out using the analytical approach outlined in section 7.1. Two different populations will be used to define the dual use categories.

##### 7.3.2.1 Modified PP population

Participants who have completed all three assessments without major protocol deviations and in whom the dual use category has remained the same across all three evaluation assessments. This category will form the basis of the analysis.

##### 7.3.2.2 PP population

Participants who have completed all three assessments without major protocol deviations. The dual use category will be assigned by calculating the mean CPD across the entire 6 month follow-up period and assessing the relative reduction versus baseline CPD, using the same categories as defined in section 8.2.

#### 7.3.3 Covariate-adjusted analysis

The allocation of patients to dual-use category for each analytical time period is predicated on the proportionate reduction in CC use from baseline. There is potential for a biased result if there is significant between-subjects variation in baseline use. For that reason, a covariate adjusted analysis (ANCOVA) will be carried out for primary and secondary outcomes, controlling for absolute CC use at baseline.

## 8 Safety analyses

All adverse events (AEs) recorded in the CRF will be fully listed, with specific attention to any events occurring post-enrollment.

A summary table will present the number of AEs categorized by severity, treatment-emergent adverse events (TEAEs), and serious adverse events (SAEs).

A TEAE is defined as an adverse event that arises during treatment, either not present before treatment or recurring after having resolved prior to treatment. Additionally, a TEAE may also be defined as an AE that worsens in severity during treatment when compared to the pre-treatment condition. If the relationship between the AE and the treatment is unclear, it will be considered treatment-related by default.

Adverse events will be coded using the Medical Dictionary for Regulatory Activities (MedDRA) and graded according to the NCI Common Terminology Criteria for Adverse Events (CTCAE), Version 5.0. The number and percentage of participants reporting TEAEs will be presented by the most severe CTCAE grade, system organ class, and preferred term, with further breakdowns by intervention group. Similarly, the number and percentage of participants reporting treatment-emergent SAEs, treatment-related AEs/SAEs, and TEAEs leading to discontinuation will be provided.

A detailed listing of AEs (including TEAEs) will be generated for each participant, including information such as the verbatim term, preferred term, system organ class, CTCAE grade, and the relationship to the study treatment. Deaths, other SAEs, and significant AEs that led to the permanent discontinuation of study treatment will also be included in this listing.

When calculating the incidence of adverse events and their subcategories based on treatment, time period, and severity, each participant will only be counted once, with any repeated occurrences of the same AE being excluded. The denominator for incidence rates will be the total population size.

A treatment-emergent adverse event (TEAE) is conventionally defined as an event that emerges during treatment, having been absent pre-treatment, or that worsens relative to the pre-treatment state. This definition is based solely on the temporal relationship to the treatment and does not depend on whether the event is considered to be an effect of the treatment. Therefore, TEAEs are a subset of the broader category of adverse events (AEs), which include all events regardless of their timing or relationship to treatment. All AEs documented in the electronic Case Report Form (eCRF) will be recorded, along with an assessment of their relationship to the treatment and any pre-treatment record of the condition. This determination will be made on a case-by-case basis during the data review process.

## 9 Reporting conventions

P-values equal to or greater than 0.001 will be presented to three decimal places, while those below 0.001 will be reported as "<0.001". The mean, standard deviation, and other summary statistics (except quantiles) will be reported with one additional decimal place compared to the original data. Quantiles, including the median and interquartile range, will be shown using the same number of decimal places as the original data.

## 10 Discussion

The majority of smoking-related diseases stem from prolonged exposure to harmful chemicals in tobacco smoke. Therefore, understanding the risks associated with the combined use of combustion cigarettes (CC) and e-cigarettes (EC) is essential. Dual users exhibit varied usage behaviors, leading to different levels of toxin exposure and health risks. By compiling a comprehensive dataset that categorizes dual-use patterns, this study aims to create a crucial resource for understanding the health implications of combined use. The methodologies outlined in this protocol are designed to better assess the risks of dual use, ensuring that both the public and policymakers have access to reliable, evidence-based information. Ultimately, the study strives to provide valuable insights for current public health decision-making and to empower consumers to make informed choices regarding available nicotine products.

This report outlines the design and statistical analysis plan for a 6-month prospective clinical trial involving 250 smokers who are interested in reducing CC use by transitioning to EC products. The primary goal is to assess the potential health effects of substituting CC with e-cigarettes, which are considered to be less harmful, by measuring various biomarkers related to the extent of cigarette replacement. By analyzing the degree of substitution, this study aims to quantify the potential risks of dual use of both CC and EC.

This research has several key strengths, including a sufficiently large sample size to allow for detailed subgroup analysis of dual users based on distinct usage patterns. It also employs a wide range of biomarkers of exposure (BoEs) and biomarkers of potential harm (BoPHs) for a comprehensive understanding. Furthermore, cigarette and e-cigarette consumption will be closely monitored through a specialized tracker app, ensuring precise data collection.

### Limitations

The development of the analytical strategy faced a number of challenges. Although the BoE used in the primary and secondary outcome analyses are well established with regard to CC use, there is more limited information available for their application in e-cigarette use and none at all in the field of dual use. It seems reasonable to assume a qualitative relationship with dual-use that falls between the two extremes of 100 % CC use and 100 % e-cigarette use, but there is insufficient prior information available to allow any quantitative assumptions to be made regarding the underlying distribution of BoE levels. For this reason, we considered the use of the non-parametric Jonckheere–Terpstra test for the primary analysis. However, although this allows the assumed trend across the categorical dual use spectrum to be captured without concerns around non-normality, it introduces other assumptions with regard to order across the groups which, if invalid, would potentially lead to a higher propensity to type II errors. We therefore reverted to an ANOVA model, with the provision for robust sandwich variance estimators being employed to handle the anticipated unequal residual variance.

There is limited potential for confounder controlling within the analytical design. The participants are self-selected and their dual use categorization is a post hoc process that will inevitably yield between-groups heterogeneity. Some elements of this variation can be accounted for – e.g. demographics, physiological status, comorbidities, pack-years exposure, absolute quantity of EC use etc - can be documented and used in descriptive analyses, there will potentially be unknown confounders exerting an influence on the results. Whilst this is probably an acceptable limitation at this early stage in the development of understanding of the role of BoE in the dual use population, future studies building on the exploratory findings of this study will need to address the issue in a more robust fashion.

As outlined in paragraph 4 regarding sample size, the estimated number of participants is based on the primary outcome measure. The null hypothesis being tested is that the mean urinary 2CyEMA levels across the various dual-use categories originate from the same population. This hypothesis will be evaluated separately at 1, 3, and 6 months, with dual-use categories reassigned at each interval, as needed, based on EC usage in the preceding 30 days.

In the absence of prior published data for this outcome and based on a fixed-effects analysis of variance (ANOVA) analysis, it was assumed that a medium effect size would be observed (f = 0.25) between the five groups under analysis. Based on α = 0.05 and 1-β = 0.8, we estimated a required total sample size of 200, or 40 participants per group. (Calculated using G∗Power v3.1.9.7)

The analysis plan does not leverage the within-subject correlation in the longitudinal measures - the longitudinal data is not intended to be analyzed longitudinally but cross-sectionally at each time point respectively. Although we acknowledge that this approach is less efficient and overlooks the ability to distinguish between- and within-subject effects, it would require a substantially more sophisticated statistical model, to allow for individuals migrating between dual use groups over the course of the study. Given that this is primarily an exploratory study at this stage, with no prior published work to characterize this migration, we did not feel confident to adopt this as an a priori analytical approach.

One issue that will require consideration in the interpretation of the results relates to the metric used to define dual-use. Participant use of CC/e-cigarettes is determined by protocol in this study, but rather reflects the wish of the individual with regard to the balance of use over the course of the study. Given that the major issue of concern is the potential for harm associated with CC use, we took the decision to define our dual-use categories in terms of relative reduction in CC use from baseline, regardless of the actual amount of vaping that the participant undertakes. This means that two individuals that reduce their CC use by 80 %, will be considered in the same dual use category, regardless of whether they achieve this with 10 EC uses per day, or 50 per day. The inevitable result of this will be a degree of heterogeneity within the categories - although estimates of actual e-cigarette usage will be documented, this information will not inform the primary and secondary analyses of the results. Whilst future research may well want to explore this issue, at this stage our objective is to confirm and calibrate the value of the markers in a dual-use population, rather than to draw broader conclusions around the optimum usage profile.

Finally, it is worth considering the analytical strategy adopted for the exploratory analyses of biomarkers of potential harm (BoPH). Although this study has a longer duration of follow-up than most previously published biomarker studies, a period of six months dual-use may nonetheless be insufficient to capture clinically relevant changes in BoPH. There is certainly likely to be a longer lag time between changes in exposure to e-cigarettes and corresponding alterations in markers compared to biomarkers of exposure (BoE). Although this is only an exploratory outcome, in order to minimize the risk of a Type II error, we have chosen to aggregate all CC use data from throughout the six-month study period, rather than assessing the outcomes for multiple sequential one-month periods (0–1 month; 2–3 months, 5–6 months), as is the case for the primary and secondary outcome analyses. This approach, whilst pragmatic, opens us up to another problem. If a substantial number of participants shift between dual-use categories over the course of the study, there is a risk that the BoPH analyses will lack adequate power. We have addressed this by analysis the BoPH changes based on two distinct study populations: firstly, the group whose dual use category remains constant across the six-month period and secondly by assessing the overall six-month CC exposure, disregarding changes over the course of the study. Whilst the first approach may be expected to yield the cleanest estimate of the impact of dual use on BoPH, it also has a high risk of participant attrition, with a consequent effect on the uncertainty associated with the estimate of effect. The second approach, whilst “noisier” from the point of view of data quality, maximizes the use of the available data and consequently gives the greatest chance of generating informative results.

The biomarkers of exposure investigated in this study are supposed to show a clear dose-response related to CPD as shown earlier by Scherer et al. [[31], [32], [33]].

In terms of BoPH, previous work showed a significant decrease in smokers after quitting within 1 week to three months for eicosanoids, such as 2,3-d-TXB2 or LTE4 [34]. Therefore, we expect to see a difference in BoPH levels after switching, if any within a few weeks to 3 months. Only light or even moderate reduction of CPD may not result in a significant decrease in BoPH levels. It is important to note here, that BoPH levels between healthy smokers and non-smoker only differ by approx. 5–25 %, however this difference is significant. Whereas the levels of biomarkers of exposure differ by a factor of 2-fold to several orders of magnitude with a significant decrease in BoE levels after quitting within 1–3 days in general.

## CRediT authorship contribution statement

**Jonathan Belsey:** Writing – review & editing, Writing – original draft, Methodology, Data curation, Conceptualization. **Jakub Weglarz:** Writing – review & editing, Project administration. **Max Scherer:** Writing – review & editing, Validation, Conceptualization. **Nikola Pluym:** Writing – review & editing, Validation, Conceptualization. **Riccardo Polosa:** Validation, Supervision, Project administration, Methodology, Conceptualization.

## Disclaimer

This investigator-led research is sponsored by ECLAT Srl, a spin-off company of the University of Catania, and funded by a competitive grant from Global Action to End Smoking (GAES), an independent U.S. nonprofit 501(c)(3) organization. GAES had no involvement in the study's design, execution, data analysis, or interpretation of the findings. The content, selection, and presentation of the information, along with any opinions expressed, are entirely the responsibility of the authors and do not necessarily reflect the views of GAES. ECLAT Srl is a research-driven company dedicated to addressing global health challenges, with a focus on harm reduction and technological innovation.

## Funding

This project received funding from the Global Action to End Smoking (GAES) Inc., a U.S.-based nonprofit private foundation with 501(c)(3) status.

## Declaration of competing interest

The authors declare the following financial interests/personal relationships which may be considered as potential competing interests:Riccardo Polosa reports financial support was provided by The 10.13039/100019021Foundation for a Smoke-Free World. If there are other authors, they declare that they have no known competing financial interests or personal relationships that could have appeared to influence the work reported in this paper.

## Data Availability

Hereby presented manuscript is an SAP (Statistical Analyses Plan) for a study that has not been carried out yet and therefore includes no data within it. All data and Code (if applicable) will be available once the study will be finished.
