# Supplementary material for: Statistical analyses plan for “MAGnItude of cigarette substitutioN after initiation oF e-cigarettes and its ImpaCt on biomArkers of exposure and potenTial harm in dual users”: MAGNIFICAT trial
Source: Heliyon. 2024 Oct 22;10(21):e39695. doi: 10.1016/j.heliyon.2024.e39695 (PMC11565015; doi:10.1016/j.heliyon.2024.e39695)
Supplement: Multimedia component 1 [file mmc1.docx]

**INFORMACJA DLA UCZESTNIKA**

**I FORMULARZ ŚWIADOMEJ ZGODY**

**INFORMACJA DLA UCZESTNIKA**

| **Badanie wskaźnika zastąpienia konwencjonalnych papierosów przez e-papierosy i jego wpływ na biomarkery narażenia oraz ocena potencjalnych szkód zdrowotnych u użytkowników obu rodzajów papierosów**  **Kod eksperymentu medycznego: P-0101** |
| --- |

**Numer kwalifikacyjny Uczestnika: |____|____|____|____|____|**

| **Ośrodek Kliniczny/Kontakt telefoniczny:** | PRATIA S.A.  MTZ Clinical Research powered by Pratia  ul. Gładka 22, 02-172 Warszawa Polska  Telefon: +48 22 572 59 59  (od poniedziałku do piątku w godz. 9:00–16:00)  Faks: +48 22 572 59 57 |
| --- | --- |
| **Główny Badacz:** | Lekarz Piotr Sobieraj, doktor nauk medycznych  PRATIA S.A.  MTZ Clinical Research powered by Pratia  ul. Gładka 22, 02-172 Warszawa Polska  Telefon: +48 22 572 59 59  Faks: +48 22 572 59 57 |
| **Kontakt w nagłych przypadkach** | **Lekarz Piotr Sobieraj**  **Tel. kom.: +48 508 825 956**  do zgłaszania poważnych sytuacji medycznych |
| **Sponsor:** | **ECLAT srl**  **Spin-off Uniwersytetu w Katanii**  Via S. Sofia 89  95123 Catania, Włochy |
| **Laboratorium Bioanalityczne** | **ABF Analytisch-Biologisches Forschungslabor GmbH**  Semmelweisstraße 5  82152 Planegg, Niemcy |
|  |  |

1. **Wprowadzenie**

Został/a Pani/Pan zaproszona/y do udziału w eksperymencie medycznym. Niniejszy dokument zawiera informacje o przebiegu i celu tego eksperymentu medycznego oraz o ryzyku i korzyściach związanych z uczestnictwem w nim. Przed wyrażeniem zgody na udział w tym eksperymencie medycznym ważne jest, aby zrozumiał/a Pani/Pan, na czym ono polega. **Prosimy uważnie przeczytać poniższe informacje i zwrócić się do Lekarza badania, określanego mianem Głównego Badacza, w celu wyjaśnienia ewentualnych wątpliwości.** Może Pani/Pan zapytać Lekarza-Badacza o wszystko, co jest, w Pani/Pana opinii, związane z eksperymentem medycznym. Przed podjęciem decyzji będzie Pani/Pan miał/a dość czasu, aby przedyskutować tę sprawę
z przyjaciółmi i z rodziną (jeśli wyrazi Pani/Pan taką chęć).

Ważne jest, aby podejmując decyzję o udziale w eksperymencie medycznym, akceptował/a Pani/Pan jego przebieg i cel oraz był/a gotowy/a uczestniczyć w nim aż do jego zakończenia.

Sponsor ECLAT srl pokrywa wszystkie koszty związane z eksperymentem medycznym. Eksperyment medyczny zostanie przeprowadzony przez PRATIA S.A., MTZ Clinical Research powered by Pratia zgodnie z wymaganiami „Dobrej Praktyki Klinicznej” (ICH GCP) oraz z zasadami etycznymi zawartymi w Deklaracji Helsińskiej i jej uzupełnieniach, a także zgodnie z polskim prawem. Ten eksperyment medyczny uzyskał pozytywną opinię niezależnej Komisji Bioetycznej.

1. **Jaki jest cel eksperymentu medycznego?**

Celem tego badania jest zbadanie wpływu zastępowania tradycyjnych papierosów potencjalnie mniej szkodliwymi produktami dostarczającymi nikotynę (w szczególności e-papierosami do waporyzacji/inhalacji) w odniesieniu do ogólnego poziomu zastępowania tradycyjnych papierosów. W badaniu będzie wykonana analiza wpływu różnych poziomów zastępowania tradycyjnych papierosów przez e-papierosy i związaną z tym ekspozycję (narażenie) na substancje toksyczne.

Po inhalacji przez użytkownika, roztwór e-płynu zawierający nikotynę dostaje się do płuc w postaci aerozolu. Celem e-papierosów jest zapewnienie wystarczająco satysfakcjonującej alternatywy dla palenia tradycyjnych papierosów. Według amerykańskiej Narodowej Akademii Nauk, Inżynierii i Medycyny (US National Academies of Sciences, Engineering, and Medicine) "istnieją niezbite dowody na to, że całkowite zastąpienie tradycyjnych papierosów przez e-papierosy zmniejsza narażenie użytkowników na liczne substancje toksyczne i rakotwórcze, które są obecne w tradycyjnych papierosach". 

1. **Informacja o badanym produkcie**

Produktem wykorzystanym w badaniu jest, poza tradycyjnymi papierosami , e-papieros o nazwie handlowej KIWI Pen 2 w zestawie, który składa się z następujących elementów:

a. e-papierosa – złożonego z baterii (typu polimerowego Li-Ion, o pojemności 400 mAh), zbiornika na roztwór nikotyny, grzałki do podgrzewania roztworu i wytwarzania z niego pary nikotynowej oraz ustnika wykonanego z miękkiego materiału – bawełny i tekturowej otoczki – imitującego tradycyjny papieros.

b. Akumulatora zewnętrznego, służącego do ładowania baterii wewnętrznej e-papierosa; o pojemności 1800 mAh oraz 6.93 Wh

c. Zasobniki z płynem do e-papierosa – w postaci zasobnika o poj. 10 ml – w opakowaniu przezroczystym, poliwęglanowym (miękki plastik), oklejone etykietą identyfikacyjną oraz z nadrukami zawierającymi niezbędne informacje odnośnie składu, zagrożeń, sposobu obchodzenia się, producencie, dystrybutorze oraz pozostałe, prawem wymagane informacje; o pojemności pojedynczego zasobnika: 10 ml, stężeniu roztworu: 10 mg nikotyny/1 ml. Pozwalające na ok. 5 uzupełnień zasobnika w e-papierosie, co odpowiada 5 opakowaniom papierosów tradycyjnych, po 20 sztuk każde. Każdy uczestnik z grupy badanej, otrzyma 7 zasobników na okres 35 dni lub 4 zasobniki na okres 20 dni (w zależności od przyjętego w harmonogramie interwału). Zapewnione zostanie 3 wersji smakowych/aromatyzowanych: czysty/suchy tytoń, mięta z tytoniem, miękki tytoń (delikatny). Wszystkie odmiany cechują się tą samą mocą, pojemnością, sposobem pakowania, przechowania oraz warunkami użycia. Smaki mogą być wydawane uczestnikom wg preferencji i nie mogą być zmieniane w trakcie badania. W trakcie wizyty związanej z pobraniem nowych, zużyte zasobniki powinny być zwrócone przez uczestników.

1. **Dostępne alternatywne metody leczenia**

Jest to eksperyment medyczny przeprowadzony z udziałem zdrowych ochotnikach i nie oczekuje się żadnych korzyści z leczenia. Dlatego alternatywne metody leczenia nie mają zastosowania w tym przypadku.

1. **Możliwe niedogodności i ryzyko dla Uczestnika lub dla embrionu, płodu lub niemowlęcia karmionego piersią**

Palenie papierosów powoduje choroby płuc i serca, a także inne poważne choroby u palaczy (np. raka płuca). Nie ma bezpiecznych papierosów, a najlepszym sposobem dla palaczy, aby zmniejszyć negatywne skutki zdrowotne palenia jest rzucenie palenia. Jeśli chciałaby Pani/chciałby Pan dowiedzieć się więcej o tym jak rzucić palenie, proszę zapoznać się z informacjami na stronie <https://pacjent.gov.pl/aktualnosc/dlaczego-warto-rzucic-palenie>, <https://jakrzucicpalenie.pl> by sprawdzić z jakiej formy pomocy może Pani/Pan skorzystać.

Każdy eksperyment medyczny z udziałem ludzi związany jest z pewnym zagrożeniem, np. ujawnieniem nieznanej nadwrażliwości na badany produkt lub wystąpieniem niespodziewanych działań niepożądanych. Ryzyko wystąpienia działań niepożądanych (podanych poniżej) spowodowanych udziałem w tym eksperymencie medycznym jest niskie, głównie ze względu na wcześniejszą ekspozycję na papierosy i produkty zawierające nikotynę.

Niektóre procedury, które zostaną wykonane w trakcie eksperymentu medycznego, mogą wiązać się z pewnym ryzykiem (podany poniżej), a lekarz prowadzący eksperyment może dostarczyć więcej informacji.

| **Procedury** | **Ryzyko** |
| --- | --- |
| Pobranie próbki krwi | - Dyskomfort spowodowany przez igłę i obrzęk lub zasinienie wokół miejsca pobierania krwi - Zawroty głowy i omdlenia (niezbyt często) - Niewielkie ryzyko krwiaka lub reakcji zapalnej w miejscu pobierania krwi |
| Spirometria Nieinwazyjne badanie czynności płuc | - Zawroty głowy i omdlenia (niezbyt często) |
| Test Chester Step | - Zawroty głowy i omdlenia (niezbyt często) |

Nad całością przebiegu eksperymentu medycznego oraz Pani/Pana zdrowiem i bezpieczeństwem będzie czuwał zespół przeszkolonych Lekarzy-Badaczy i Pielęgniarek. W przypadku nagłego poważnego pogorszenia Pani/Pana stanu zdrowia lub samopoczucia, wywołanego przez udział w eksperymencie medycznym, Lekarz-Badacz zapewni odpowiednie leczenie. Ośrodek wyposażony jest w niezbędny sprzęt potrzebny przy niesieniu pomocy w nagłych przypadkach, jeśli to konieczne.

**Ważne jest, aby wszelkie, wcześniej nieodczuwalne, niepokojące objawy, w razie, gdyby takie wystąpiły, były przez Panią/Pana niezwłocznie zgłaszane Lekarzowi-Badaczowi.**

**Uwaga:**

**Zatajenie lub też podawanie fałszywych informacji o stanie zdrowia w trakcie procedur kwalifikacyjnych może spowodować poważne komplikacje zdrowotne w trakcie eksperymentu medycznego.**

Wszystkie zdarzenia niepożądane będą rejestrowane, a Lekarze-Badacze i Pielęgniarki podejmą niezbędne działania zmierzające do złagodzenia ich negatywnych następstw.

**Wpływ na zdolność prowadzenia pojazdów i obsługiwania maszyn**

**Nie stwierdzono wpływu na zdolność prowadzenia pojazdów i obsługę maszyn**

**Przewidywalne ryzyko dla embrionu, płodu lub niemowlęcia karmionego piersią**

Ciąża

Takie samo jak w przypadku palenia tradycyjnych papierosów, czy używania innych produktów nikotynowych. Istnieje wysokie ryzyko uszkodzenia embrionu ludzkiego. Kobiety w ciąży nie mogą brać udziału w projekcie.

Karmienie piersią

Takie samo jak w przypadku palenia tradycyjnych papierosów, czy używania innych produktów nikotynowych. Istnieje wysokie ryzyko chorób dla dziecka karmionego piersią przez kobietę palącą e-papierosy, papierosy lub używającą innych produktów nikotynowych. Kobiety karmiące piersią nie mogą brać udziału w projekcie

Kobiety w ciąży lub karmiące piersią nie będą mogły wziąć udziału w tym eksperymencie medycznym. Jeżeli w trakcie eksperymentu zajdzie Pani w ciążę, Pani udział w eksperymencie zostanie zakończony i zostanie Pani zalecone zwrócenie się z prośbą o opiekę przedporodową
i poradę do swojego lekarza podstawowej opieki zdrowotnej. Skontaktujemy się z Panią i poprosimy o powiadomienie nas o wyniku rozwiązania ciąży.

1. **Kwalifikacja do eksperymentu medycznego**

Kwalifikacja do eksperymentu medycznego odbędzie się w trakcie 14 dni przed planowanym włączeniem do eksperymentu. W oparciu o wyniki przeprowadzonych badań i na podstawie wywiadu medycznego, Lekarze-Badacze zadecydują, czy spełnia Pani/Pan wszystkie kryteria włączenia do eksperymentu i czy nie ma żadnych przeciwwskazań do Pani/Pana udziału w eksperymencie. Jeżeli nie zostanie Pani/Pan włączona/y do badania, to Lekarz-Badacz wyjaśni Pani/Panu przyczynę tej decyzji.

Lekarze-Badacze zakwalifikują do eksperymentu jedynie osoby bez istotnych klinicznie chorób lub odchyleń w wynikach badań, na podstawie wszystkich zebranych informacji o Uczestnikach. Osoby z istotnymi odchyleniami w wynikach badań nie będą mogły wziąć udziału w eksperymencie. Nie wszyscy uczestnicy z prawidłowymi wynikami badań kwalifikacyjnych zostaną włączeni do eksperymentu.

**Aby zakwalifikować się do eksperymentu trzeba spełnić poniższe kryteria włączenia:**

1. Osoba, która przeczytała, zrozumiała i podpisała Formularz Świadomej Zgody.
2. Osoba w wieku od 19 lat w dniu wizyty kwalifikacyjnej.
3. Aktualni palacze (≥15 dziennie) wyłącznie konwencjonalnych papierosów
4. Historia regularnego palenia przez co najmniej 12 nieprzerwanych miesięcy
5. Zweryfikowany status palenia (eCO ≥ 7 ppm)
6. Chęć przejścia na produkt waporyzujący i podjęcie próby ograniczenia spożycia papierosów konwencjonalnych (tylko grupa badawcza A).
7. Odmowa próby rzucenia palenia.
8. Dobry stan fizyczny i psychiczny w opinii Badacza na podstawie wywiadu lekarskiego, parametrów życiowych (ciśnienie krwi, tętno) i spirometrii.

**W eksperymencie nie mogą uczestniczyć osoby, które spełnią chociaż jedno z poniżej wymienionych kryteriów wyłączenia z eksperymentu:**

1. Planowanie rzucenie palenia w ciągu najbliższych 30 dni.
2. Znane istotne klinicznie choroby sercowo-naczyniowe, układu oddechowego, psychiatryczne lub inne poważne choroby, które w opinii Badacza mógłby zagrażać bezpieczeństwu uczestnika lub mieć wpływ na ważność wyników eksperymentu.
3. Regularne stosowanie jakichkolwiek leków.
4. Stwierdzone w wywiadzie nadużywanie alkoholu albo narkotyków.
5. Stosowanie jakichkolwiek produktów nikotynowych (np. e-papierosy, woreczki nikotynowe) lub tytoniowych (np. podgrzewane wyroby tytoniowe, doustnie produkty bezdymne) innych niż własne konwencjonalne papierosy w ciągu 3 miesięcy od wizyty kwalifikacyjnej.
6. Stosowanie nikotynowej terapii zastępczej lub innego sposobu rzucenia palenia w ciągu 3 miesięcy przed wizytą kwalifikacyjną.
7. Kobiety w ciąży, kobiety karmiące piersią lub kobiety chcące zajść w ciążę w trakcie eksperymentu (zostanie wykonany test ciążowy z próbki moczu).
8. Aktywny udział w innym badaniu.

**OSTATECZNA DECYZJA O ZAKWALIFIKOWANIU UCZESTNIKA DO EKSPERYMENTU MEDYCZNEGO JEST PODEJMOWANA PRZEZ LEKARZA - BADACZA NA PODSTAWIE DOSTĘPNYCH INFORMACJI UZYSKANYCH W CZASIE KWALIFIKACJI.**

1. **Przebieg eksperymentu medycznego, procedury**

Badanie zostanie przeprowadzane na grupie 300 uczestników różnego wieku i płci – aktywnych palaczy, którzy dobrowolnie wyrażą chęć udziału w nim. 250 osób (Grupa A) stanowić będzie grupę badawczą, której zaproponowany zostanie produkt alternatywny do tradycyjnych papierosów. Będzie to urządzenia typu e-papieros (nazwa rynkowa: KIWI) do stosowania z wkładem płynnym, zawierającym roztwór nikotyny (10 mg/ml). Zadaniem uczestników w tej grupie będzie prowadzenie dziennika przy pomocy aplikacji dedykowanej na urządzenia mobilne, w którym będą rejestrować ilość wypalonych papierosów tradycyjnych i/lub użytych wkładów do e-papierosa w ciągu dnia poprzedniego oraz odbycie 4 wizyt w ośrodku badawczym w celu przeprowadzenia kompletu badań – zgodnie z planem poniżej. Grupa ta będzie zaopatrzona we wkłady na okres badania, które będą wydawane zgodnie z harmonogramem opisanym poniżej. Aplikacja na urządzenia mobilne będzie gromadzić jedynie odpowiedzi uczestnika, wprowadzone rano, w odniesieniu do ilości wypalonych papierosów konwencjonalnych i/lub liczby użyć e-papierosa w dniu poprzednim. Aplikacja nie będzie gromadzić żadnych innych danych o użytkowniku. Wszystkie szczegóły związane z jej działaniem oraz modułami zostały opisane w instrukcji obsługi aplikacji.

W ramach badania wyodrębniona będzie 50-osobowa grupa kontrolna (Grupa B), która pozostanie przy stosowaniu swoich tradycyjnych papierosów i przy bieżących nawykach w zakresie palenia papierosów. Zadaniem grupy kontrolnej, będzie prowadzenie dziennika w postaci codziennego uzupełnienia informacji o ilości wypalonych papierosów w dniu poprzedzającym uzupełnianie dzienniczka, w aplikacji dedykowanej na urządzenia mobilne oraz 4 wizyt w ośrodku badawczym, w celu przeprowadzenia kompletu badań – zgodnie z planem poniżej.

Całość badania dla każdego uczestnika będzie trwała około 6 miesięcy.

**SCHEMAT EKSPERYMENTU MEDYCZNEGO**

| **WIZYTA KWALIFIKACYJNA**  1-14 dni przed pierwszym zastosowaniem badanego produktu | - Identyfikacja Uczestnika (konieczny dowód tożsamości lub inny dokument ze zdjęciem). - Zapoznanie się z Informacją dla Uczestnika i podpisanie Formularza Świadomej Zgody na uczestnictwo w badaniu w obecności Lekarza-Badacza. - Wywiad medyczny, demograficzny i dotyczący stylu życia, w tym historia palenia papierosów. ^1^ - Deklaracja, czy uczestnik chce testować e-papierosy, czy decyduje się na kontunuowanie jedynie swoich własnych papierosów. - Pomiar poziomu wydychanego tlenek węgla - Pomiar ciśnienie krwi i tętna - Spirometria - Test ciążowy z moczu, dla kobiet - Wydanie zestawu do zbiórki moczu, na następną wizytę |
| --- | --- |

⇓

| **Wizyta 1** (Dzień 0) | - Potwierdzenie kryteriów włączenia / wyłączenia do badania - Wywiad dotyczący palenia papierosów. - Pomiar poziomu wydychanego tlenek węgla - Pomiar ciśnienie krwi i tętna - Pomiar wagi i wzrostu (wyliczenie BMI) - Kwestionariusze, związane z paleniem papierosów - Odebranie próbki moczu, pobranej rano, w domu przez uczestnika. - Wydanie zestawu do zbiórki moczu, na następną wizytę - Test ciążowy z moczu, dla kobiet - Chester step test (VO2max) - Pobranie próbki krwi - Instalacja aplikacji na telefon komórkowy uczestnika badania, wraz ze szkoleniem z obsługi. - Raportowanie zdarzeń niepożądanych   **Poniżej procedury TYLKO DLA GRUPY A**  **(dla osób, które zdecydowały się na testowanie e-papierosów)**   - Zaznajomienie się z e-papierosem - Wydanie produktów do waporyzacji |
| --- | --- |

⇓

| **Dzień 14** | **TYLKO DLA GRUPY A**  **(dla osób, które zdecydowały się na testowanie e-papierosów)**  Odbiór zużytych i niezużytych zasobników z płynem, wydanie uczestnikom nowych zasobników na kolejny okres |
| --- | --- |

⇓

| **Wizyta 2** (Dzień 28) | - Wywiad dotyczący palenia papierosów. - Kwestionariusze, związane z paleniem papierosów - Pomiar poziomu wydychanego tlenek węgla - Pomiar ciśnienie krwi i tętna - Pobranie próbki krwi - Pomiar wagi (wyliczenie BMI) - Odebranie próbki moczu, pobranej rano, w domu przez uczestnika. - Wydanie zestawu do zbiórki moczu, na następną wizytę - Test ciążowy z moczu, dla kobiet - Spirometria - Chester step test (VO2max) - Raportowanie zdarzeń niepożądanych   **Poniżej procedury TYLKO DLA GRUPY A**  **(dla osób, które zdecydowały się na testowanie e-papierosów)**   - Sprawdzenie stosowania z e-papierosa - Odbiór zużytych i niezużytych zasobników z płynem, wydanie uczestnikom nowych zasobników na kolejny okres |
| --- | --- |

⇓

| **Dzień 63** | **TYLKO DLA GRUPY A**  **(dla osób, które zdecydowały się na testowanie e-papierosów)**  Odbiór zużytych i niezużytych zasobników z płynem, wydanie uczestnikom nowych zasobników na kolejny okres |
| --- | --- |

⇓

| **Wizyta 3** (Dzień 91) | - Wywiad dotyczący palenia papierosów. - Kwestionariusze, związane z paleniem papierosów - Pomiar poziomu wydychanego tlenek węgla - Pomiar ciśnienie krwi i tętna - Pobranie próbki krwi - Pomiar wagi (wyliczenie BMI) - Pobranie próbki wydychanego powietrza - Odebranie próbki moczu, pobranej rano, w domu przez uczestnika. - Wydanie zestawu do zbiórki moczu, na następną wizytę - Test ciążowy z moczu, dla kobiet - Spirometria - Chester step test (VO2max) - Raportowanie zdarzeń niepożądanych   **Poniżej procedury TYLKO DLA GRUPY A**  **(dla osób, które zdecydowały się na testowanie e-papierosów)**   - Sprawdzenie stosowania z e-papierosa - Odbiór zużytych i niezużytych zasobników z płynem, wydanie uczestnikom nowych zasobników na kolejny okres |
| --- | --- |

⇓

| **Dzień 119** | **TYLKO DLA GRUPY A**  **(dla osób, które zdecydowały się na testowanie e-papierosów)**  Odbiór zużytych i niezużytych zasobników z płynem, wydanie uczestnikom nowych zasobników na kolejny okres |
| --- | --- |

⇓

| **Dzień 154** | **TYLKO DLA GRUPY A**  **(dla osób, które zdecydowały się na testowanie e-papierosów)**  Odbiór zużytych i niezużytych zasobników z płynem, wydanie uczestnikom nowych zasobników na kolejny okres |
| --- | --- |

⇓

| **Wizyta 4** (Dzień 182) | - Wywiad dotyczący palenia papierosów. - Kwestionariusze, związane z paleniem papierosów - Pomiar poziomu wydychanego tlenek węgla - Pomiar ciśnienie krwi i tętna - Pobranie próbki krwi - Pomiar wagi (wyliczenie BMI) - Odebranie próbki moczu, pobranej rano, w domu przez uczestnika. - Test ciążowy z moczu, dla kobiet - Spirometria - Chester step test (VO2max) - Raportowanie zdarzeń niepożądanych   **Poniżej procedury TYLKO DLA GRUPY A**  **(dla osób, które zdecydowały się na testowanie e-papierosów)**   - Sprawdzenie stosowania z e-papierosa - Odbiór zużytych i niezużytych zasobników z płynem |
| --- | --- |

^1^Zostanie Pani/Pan zapytana/y m.in. o obecny stan zdrowia, przebyte choroby i operacje, przyjmowane leki wydawane na receptę i bez recepty oraz suplementy diety. Pytania będą także dotyczyć stylu życia, w tym: sposobu żywienia, palenia papierosów, ilości spożywanego alkoholu, aktywności fizycznej i udziału w innych badaniach klinicznych.

**Wykonywane będą wymienione badania:**

| **Badania krwi** | **Badanie moczu** |
| --- | --- |
| - Akroleina (3-HPMA) - 1,3-butadien (MHBMA) - Tlenek propylenu (2-HPMA) - Aldehyd krotonowy (HMPMA) - Benzen (SPMA) - Styren (PHEMA) - Glicydol (DHPMA) - Izopren (IPMA) - Toluen (SBMA) - Tlenek etylenu (HEMA) - Akrylonitryl (CEMA/CeVal) - Akryloamid (AAMA/GAMA/GlyVal) - Metabolity węglowodorów poliaromatycznych (benzo[a]piren, piren, fenantren, naftalen) - Aminy aromatyczne (3-/4-aminobifenyl, 2-aminonaftalen, orto-toluidyna) - całkowitych równoważników nikotyny - (metylonitrozoamino)-1-(3-pirydylo)-1-butanol (NNAL) - N-nitrosonornikotyna (NNN) - Glikol propylenowy - rozpuszczalna cząsteczka adhezji międzykomórkowej 1 (sICAM-1) w osoczu, - czynnik różnicowania wzrostu 15 (GDF-15) w osoczu - adduktom hemoglobiny | • Eikozanoidy w moczu  Kreatynina w moczu |

**Pobieranie próbek krwi**

Pobieranie próbek krwi będzie obejmować po około 10 ml krwi do badań biomarkerów pobranej podczas wizyt 1, 2, 3, 4. Dlatego oczekuje się, że całkowita objętość pobieranej krwi wyniesie  około 40 ml w przypadku każdego uczestnika podczas badania.

Zebrane próbki krwi będą analizowane przy użyciu sprawdzonych metod w odpowiednio wyposażonym laboratorium. Wyznaczone laboratorium bioanalityczne będzie odpowiedzialne za przechowywanie Pani/Pana próbek w tym okresie oraz za ich późniejsze zniszczenie po podpisaniu raportu z eksperymentu medycznego.

Próbki pobrane od Pani/Pana będą wykorzystywane wyłącznie do celów eksperymentu medycznego i bez Pani/Pana uprzedniej zgody i zatwierdzenia przez Komisję Bioetyczną nie będą wykonywane żadne inne analizy poza analizami związanymi z badaniem opisanymi w niniejszym dokumencie informacyjnym.

**Pobieranie próbek moczu**

Podczas eksperymentu medycznego będą zbierane jednorazowe próbki moczu do ogólnego badania moczu w ramach oceny bezpieczeństwa, w przypadku kobiet, również do wykonania testów ciążowych.

Uczestnik musi pobrać próbkę moczu w domu, z pierwszej próbki oddawanej danego dnia i przynieść do Ośrodka na wizytę,

1. **Gratyfikacja finansowa**
2. Uczestnictwo w eksperymencie medycznym nie będzie wiązało się dla Pani/Pana z żadnymi kosztami. Produkt badawczy zostanie dostarczony bezpłatnie i nie zostanie Pani/Pan obciążony/a żadnymi opłatami za żadne procedury wykonane w ramach tego eksperymentu.
3. Udział w eksperymencie medycznym nie zapewni Pani/Panu bezpośrednich korzyści zdrowotnych. Decydując się jednak na udział w nim, pomaga Pani/Pan w uzyskaniu ważnych informacji na temat badanego produktu.
4. Korzyścią dla Pani/Pana będzie dokładna ocena lekarska Pani/Pana stanu zdrowia przed i po eksperymencie, a kopie wyników (i opisów) badań laboratoryjnych, spirometrii otrzyma Pani/Pan po zakończeniu eksperymentu, jeśli wyrazi Pani/Pan takie życzenie.
5. Celem zrekompensowania Pani/Panu czasu, który poświęci Pani/Pan na dojazdy do Ośrodka, pobyt w nim w czasie eksperymentu i innych niedogodności związanych z uczestnictwem, otrzyma Pani/Pan gratyfikację finansową w wysokości przedstawionej poniżej.

**Uwaga: gratyfikacja finansowa będzie przysługiwała TYLKO osobom zakwalifikowanym do eksperymentu i które odbyły wszystkie wizyty zgodne z planem eksperymentu.** **Udział w wizycie kwalifikacyjnej NIE wiąże się z żadną gratyfikacją pieniężną.**

Z zakwalifikowanymi Uczestnikami zostanie zawarta umowa cywilno-prawna (umowa zlecenia). Osoby, zarejestrowane w Urzędzie Pracy jako bezrobotne, będą musiały się wyrejestrować na czas trwania umowy. Osoby nieobjęte obowiązkowym ubezpieczeniem społecznym zostaną nim objęte na czas trwania umowy (składka zostanie opłacona przez ośrodek kliniczny).

Obcokrajowcy są zobowiązani do przedstawienia stosowne dokumenty uprawniającego ich do pracy w Polsce.

Gratyfikacja finansowa za uczestnictwo w eksperymencie zostanie wypłacona przelewem bankowy w ciągu 14 dni po zakończeniu wszystkich procedur z nim związanych. Wysokość gratyfikacji jest następująca:

- 2 100 PLN brutto dla grupy A za uczestnictwo w całym eksperymencie medycznym.
- 1 000 PLN brutto dla grupy B za uczestnictwo w całym eksperymencie medycznym.
- 200 PLN brutto za umieszczenie Pani/Pana na liście rezerwowej i oczekiwanie w Ośrodku od rana do końca wizyty 1 w Dniu 0. Jeśli uczestnicy z grupy rezerwowej wezmą udział
  w eksperymencie, otrzymają taką samą gratyfikację jak uczestnicy z grupy głównej nie otrzymają dodatkowej gratyfikacji za znalezienie się na liście rezerwowej.
- 100 PLN brutto w przypadku zrezygnowania z udziału w eksperymencie, do czego przysługuje Pani/Panu prawo w każdym momencie, bez konieczności podania przyczyny.
- Jeśli przyczyną przerwania udziału będzie brak przestrzegania przez Panią/Pana obowiązków i ograniczeń związanych z eksperymentem lub niepodporządkowanie się Regulaminowi PRATIA S.A. MTZ Clinical Research powered by Pratia, poleceniom Lekarza-Badacza i personelu Ośrodka nie otrzyma Pani/Pan żadnej gratyfikacji finansowej za udział w eksperymencie, niezależnie od momentu w którym zostali wykluczeni z eksperymentu.
- Jeśli Pani/Pana udział w eksperymencie zostanie przerwany ze względów zdrowotnych po zastosowaniu badanego produktu, otrzyma Pani/Pan gratyfikację finansową proporcjonalną do udziału w eksperymencie - 250 PLN brutto za dzień wizyty.
- Uczestnicy zakwalifikowani do eksperymentu ale wykluczeni przez Badacza z powodów medycznych przed użyciem produktu badanego, otrzymają wynagrodzenie takie jak uczestnicy z grupy rezerwowej.

1. **Informacje ogólne**

**9.1. Ubezpieczenie**

Zgodnie z aktualnymi wymogami Ośrodek Badań Klinicznych wraz z personelem przeprowadzającym eksperyment medyczny objęty jest ubezpieczeniem odpowiedzialności cywilnej stosownie do polisy wystawionej przez Towarzystwo Ubezpieczeń i Reasekuracji WARTA S.A. rondo I. Daszyńskiego 1, 00-843 Warszawa**,** numer polisy: **908211598187**.

Ubezpieczenie obejmuje odpowiedzialność cywilną Ośrodka Badań Klinicznych przeprowadzającego eksperyment medyczny za szkody wyrządzone jego działaniem lub zaniechaniem uczestnikowi eksperymentu medycznego (np. uszkodzenie ciała, rozstrój zdrowia lub śmierć uczestnika eksperymentu, związane z eksperymentem i powstałe w czasie trwania ochrony ubezpieczeniowej). Roszczenia może Pani/Pan zgłaszać bezpośrednio do ubezpieczyciela. Kwota odszkodowania zostanie ustalona zgodnie z polskim prawem cywilnym. Główny Badacz posiada kopię tej polisy i warunków ubezpieczenia, które są dostępne do wglądu.

**9.2. Wycofanie się z eksperymentu medycznego**

Udział w eksperymencie medycznym jest całkowicie dobrowolny i w każdej chwili możliwe jest wycofanie się Pani/Pana z dalszego w nim udziału bez podawania przyczyny. Odmowa wzięcia udziału w eksperymencie lub wycofanie się z eksperymentu nie pociągną za sobą żadnej szkody ani utraty korzyści, do jakich jest Pani/Pan z innych względów uprawniona/y. W żaden sposób nie wpłynie to na przysługującą Pani/Panu opiekę medyczną w przyszłości. W przypadku podjęcia decyzji o wycofaniu się z eksperymentu uprzejmie prosimy o niezwłoczne powiadomienie o tym fakcie Lekarza-Badacza prowadzącego eksperyment. W przypadku pojawienia się jakichkolwiek niepokojących objawów, może Pani/Pan zostać poproszona/y o ponowne zgłoszenie się do Ośrodka na dodatkową wizytę ambulatoryjną lub Lekarz-Badacz może skierować Pana/Panią do lekarza rodzinnego lub specjalisty.

W przypadku niechęci lub niemożności pojawienia się w Ośrodku uprzejmie prosimy o telefoniczne przekazanie informacji na temat Pani/Pana stanu zdrowia, bo wszelkie zdarzenia niepożądane mogą mieć znaczenie dla innych Uczestników, a także dla analizy wyników eksperymentu.

Należy podkreślić, że brak formalnych następstw wycofania się z eksperymentu jest niezależny od faktycznych ograniczeń, wynikających z istoty stosowania produktu badanego. Zatem nawet w razie przedwczesnego zakończenia eksperymentu należy przestrzegać ograniczeń wymienionych wyżej w sekcji „Ograniczenia i wymogi związane z udziałem w eksperymencie medycznym”.

**9.3. Zakończenie Pani/Pana udziału w eksperymencie medycznym**

Możliwe przyczyny:

- Wycofanie zgody przez Uczestnika (jest Pani/Pan do tego uprawniona/y w każdym momencie eksperymentu medycznego bez konieczności podawania przyczyny).
- Uczestnik wymaga regularnego podania jakiegokolwiek leku, o którym wiadomo lub podejrzewa się, że ma wpływ na metabolizm, a przez to na stężenie badanych substancji we krwi – decyzję podejmuje Sponsor oraz Lekarz-Badacz w Ośrodku.
- Wszelkie inne istotne klinicznie nieprawidłowe wartości laboratoryjne.
- Wystąpienie jakiegokolwiek zdarzenia medycznego uniemożliwiającego dalszy bezpieczny udział Uczestnika w eksperymencie medycznym (np.: powikłania, zdarzenie/a niepożądane, podejrzenie zajścia w ciążę).
- Znaczne trudności w uzyskaniu próbek krwi, jeśli zostanie uznane, że ma to znaczący wpływ na ocenę profilu stężeń badanych substancji.
- Podejrzenie lub udowodnienie, że Uczestnik mimo wcześniejszej kwalifikacji, nie spełnia kryteriów włączenia do eksperymentu.
- Ciąża i/lub karmienie piersią u Uczestniczki eksperymentu.
- Uzasadnione podejrzenie, że Uczestnik nie przestrzega obowiązków związanych z eksperymentem (np.: nie uczestniczy w procedurach badania, nie wypełnia dzienniczka w aplikacji). Wyłączenie Uczestnika zależy od decyzji Lekarza-Badacza.
- Inny powód, który w opinii Lekarza-Badacza uniemożliwia dalszy bezpieczny udział Uczestnika w eksperymencie.

W przypadku wcześniejszego zakończenia udziału w eksperymencie przeprowadzona zostanie z Panią/Panem rozmowa z lekarzem prowadzącym eksperyment. Wszelkie dodatkowe badania lub wizyty w Ośrodku Badań Klinicznych będą przeprowadzane tylko wtedy, gdy będzie to wskazane po zapoznaniu się z opinią lekarza prowadzącego eksperyment. W przypadku wcześniejszego zakończenia udziału w eksperymencie, uczestnicy, którzy otrzymali produkty badanie do domu, powinni je zwrócić do ośrodka.

*Dotyczy kobiet w wieku rozrodczym*

W przypadku zajścia przez Panią w ciążę, prosimy o zgodę na zbieranie informacji medycznych na temat ciąży, jej przebiegu oraz, w stosownych przypadkach, narodzin i zdrowia dziecka (do 45 dni po porodzie). Może Pani podać te informacje samodzielnie lub wyrazić zgodę na to, aby Pani lekarz opieki zdrowotnej przekazał je bezpośrednio Lekarzowi - Badaczowi. Celem gromadzenia tych informacji jest ustalenie, w jaki sposób badany produkt może wpłynąć na płód.

Badacz przekaże te informacje Sponsorowi eksperymentu. Zostanie poddane ocenie, czy istnieje jakiekolwiek ryzyko dla Pani lub Pani nienarodzonego dziecka, i poinformują Panią, a w przypadku wyrażenia na to zgody, także lekarza rodzinnego o zajściu w ciążę podczas udziału w eksperymencie.

- 1. **Przerwanie eksperymentu medycznego**

Eksperyment medyczny może zostać przerwany przez Sponsora w dowolnym momencie jego trwania, w przypadku uzyskania nowych danych o bezpieczeństwie stosowanego produktu lub w przypadku, gdyby liczba lub nasilenie zdarzeń niepożądanych w opinii Głównego Badacza mogła wpłynąć na bezpieczeństwo Uczestników. W przypadku wystąpienia takiej sytuacji zostanie Pani/Pan o tym poinformowana/y.

**9.5. Co się dzieje z Pani/Pana próbkami**

Próbki krwi zostaną przesłane do wyznaczonego laboratorium bioanalitycznego zakontraktowanego przez Sponsora. Pani/Pana dane osobowe, nie zostaną umieszczone na próbkach, będą one oznaczone numerem Uczestnika badania, który zostanie Pani/Panu przypisany na początku badania.

Próbki krwi do badań laboratoryjnych będą przechowywane przez około 4 miesiące po wykonaniu analizy, a następnie zostaną zniszczone.

Jeśli wycofa Pani/Pan zgodę na udział w badaniu, Pani/Pana próbki mogą być nadal wykorzystywane do bieżących badań. Jeśli nie chce Pani/Pan, aby Pani/Pana próbki były wykorzystywane do bieżących badań, może się Pani/Pan skontaktować z lekarzem prowadzącym badanie i zlecić zniszczenie próbek, jeśli nie zostały one jeszcze wykorzystane. Jakiekolwiek wykorzystanie Pana/Pani próbek, w tym informacji przetworzonych z Pani/Pana próbek, które nastąpi przed wycofaniem przez Panią/Pana zgody, zostanie wykorzystane przez Sponsora w celu zachowania integralności badań.

**9.6. Uzyskiwanie dodatkowych informacji**

Jeżeli ma Pani/Pan jakiekolwiek pytania związane z niniejszym eksperymentem medycznym, zakresem praw, jak również zgłaszaniem ewentualnych szkód powstałych w związku
z uczestnictwem w eksperymencie, w tym jakichkolwiek niepokojących objawów zdrowotnych podczas trwania eksperymentu, bardzo prosimy o przedstawienie ich lekarzowi prowadzącemu eksperyment. Możliwy jest także kontakt z Głównym Badaczem – lekarz Piotr Sobieraj, PRATIA S.A. MTZ Clinical Research powered by Pratia, tel.: **+48 22 572 59 59**, faks: +48 22 572 59 57 (w dni robocze od 9:00 – 16:00), po godzinach pracy lub dni wolne pod tel. kom.: **+48 508 825 956** – tylko do zgłaszania poważnych sytuacji medycznych.

W przypadku pojawienia się wszelkich dodatkowych informacji na temat eksperymentu mogących mieć wpływ na wolę dalszego uczestnictwa w eksperyment, Lekarz-Badacz zobowiązuje się do ich niezwłocznego przekazania Uczestnikowi.

Osoby, które zostały zakwalifikowane do eksperymentu lub już są jego uczestnikami, posiadają uprawnienia, które wynikają z Praw Pacjenta zamieszczonych w Karcie Praw Pacjenta. Informacji na ten temat może udzielić powołane przez Ministra Zdrowia Biuro Rzecznika Praw Pacjenta/Uczestnika pod numerem infolinii 800 190 590. Infolinia jest telefonem bezpłatnym, czynnym od poniedziałku do piątku w godzinach 8:00 – 18:00.

**9.7. Obowiązki Uczestnika**

Obowiązkiem Uczestnika jest postępowanie zgodne z podanymi w Informacji dla Uczestnika wymogami i ograniczeniami eksperymentu, stosowanie się do zaleceń personelu Ośrodka Klinicznego oraz traktowanie innych współuczestników eksperymentu z szacunkiem, udzielanie rzetelnych informacji związanych z uczestnictwem w eksperymencie oraz punktualne zgłaszanie się do Ośrodka. W trakcie pobytu w Ośrodku Uczestnicy są zobowiązani do przestrzegania Regulaminu Ośrodka Klinicznego.

1. **Poufność i ochrona danych osobowych i medycznych**

ECLAT srl (Sponsor) zarejestrowany pod adresem: Via S. Sofia 89, 95123 Catania, Włochy, jest Administratorem Danych, co oznacza odpowiedzialność za wszystkie dane osobowe zebrane od Pani/Pana podczas eksperymentu medycznego oraz za dopilnowanie, aby wszystkie osoby pracujące nad eksperymentem spełniały wszelkie wymogi ochrony danych w zakresie gromadzenia, wykorzystywania i przetwarzania danych osobowych zgromadzonych w ramach tego eksperymentu.

Sponsor jest odpowiedzialny za podjęcie decyzji, jakie dane osobowe muszą być gromadzone podczas eksperymentu i w jaki sposób te dane będą wykorzystywane.

Lekarz prowadzący eksperyment i personel ośrodka badawczego będą odpowiedzialni za zbieranie danych osobowych, zgodnie z wymaganiami, w celu umożliwienia Pani/Panu udziału w eksperymencie. Oprócz danych medycznych (w tym danych z próbek laboratoryjnych) inne gromadzone dane mogą obejmować płeć, wiek lub datę urodzenia, pochodzenie etniczne, masę ciała i wzrost. Pani/Pana dane osobowe związane z udziałem w eksperymencie zostaną zastąpione kodem pseudonimizującym dane, w celu zachowania pełnej anonimowości osoby, od której je uzyskano. Tylko lekarz prowadzący eksperyment i personel ośrodka badawczego będą mogli zidentyfikować Panią/Pana na podstawie kodu i tylko w okresie niezbędnym do celów przeprowadzenia eksperymentu i uzyskania danych z eksperymentu. Sponsor i inne firmy współpracujące ze Sponsorem w eksperymencie (przedstawiciele Sponsora) nie będą w stanie Pani/Pana zidentyfikować.

Sponsor i jego przedstawiciele będą odpowiedzialni za przetwarzanie uzyskanych informacji, które będą przechowywane zgodnie z przydzielonym Pani/Panu kodem oraz będą odpowiedzialni za zapewnienie zachowania poufności danych, zgodnie z wymogami prawa w Pani/Pana kraju. Pani/Pana dane osobowe, będące w posiadaniu Sponsora lub jego przedstawicieli, będą przechowywane przez okres do 25 lat po zakończeniu eksperymentu i tylko w formie spseudonimizowanej.

Ze względu na Pani/Pana bezpieczeństwo, dostęp do niezakodowanych danych będą miały podmioty uprawnione do przeprowadzenia monitorowania, audytu i kontroli badań.

W zakresie dozwolonym przez obowiązujące przepisy prawne, niektórzy upoważnieni przedstawiciele Sponsora (w tym organizacja prowadząca eksperyment współpracująca ze Sponsorem, laboratoria testujące próbki) i przedstawiciele Komisji Bioetycznej będą mieli ograniczony dostęp do Pani/Pana danych osobowych (zapisów medycznych) przechowywanych przez ośrodek badań klinicznych. Nastąpi to w przypadku, gdy jest to wymagane do sprawdzenia prawidłowości przeprowadzonych procedur i uzyskanych danych, ale informacje te pozostaną poufne zgodnie z wymogami prawa.

W przypadku publikacji przez Sponsora [lub Lekarza prowadzącego eksperyment] jakichkolwiek wyników eksperymentu, zostanie zachowana Pani/Pana anonimowość.

Ma Pani/Pan określone prawa do dostępu i poprawy wszelkich nieprawidłowości w informacjach zebranych na Pani/Pana temat.

Bez względu na okoliczności ma Pani/Pan prawo sprzeciwić się przetwarzaniu danych, prawo do przenoszenia danych oraz prawo do uzyskania informacji o przekazaniu danych osobowych.

Może Pan/i zażądać ograniczenia wykorzystana Pan/Pan danych osobowych lub ich usunięcia.

Jeżeli przetwarzanie danych osobowych wymaga Pani/Pana zgody, ma Pani/Pan prawo do wycofania zgody w dowolnym momencie, jednak bez wpływu na zgodność z prawem przetwarzania opartego na zgodzie przed takim wycofaniem. Aby skorzystać z tych praw, należy złożyć pisemne podanie do Sponsora. Można to zrobić, składając wniosek za pośrednictwem lekarza z Ośrodka, który przekaże Pani/Pana żądanie Sponsorowi. W przypadku decyzji o zakończeniu udziału w eksperymencie, żadne dodatkowe dane osobowe nie będą gromadzone. Wszelkie dane osobowe zebrane do momentu wycofania się z eksperymentu będą zachowane i wykorzystane, ponieważ stanowią część danych eksperymentu.

W przypadku podejrzenia, że którekolwiek z Pani/Pana praw związanych z gromadzeniem lub wykorzystaniem Pani/Pana danych osobowych zostało naruszone, należy skontaktować się z przedstawicielem Sponsora pod adresem mailowym info@eclatrbc.it . Jeżeli Pani/Pana obawy nie zostaną rozwiane w sposób zadowalający, istnieje możliwość złożenia skargi do organu nadzorczego ochrony danych w Pani/Pana kraju.

Pani/Pana dane osobowe mogą być przekazywane w obrębie Europejskiego Obszaru Gospodarczego (EOG), tylko pod warunkiem podjęcia niezbędnych środków w celu zapewnienia zgodności z ogólnym rozporządzeniem o ochronie danych UE (rozporządzenie 2016/679) („RODO”) oraz krajowych lub obowiązujących przepisów prawa dotyczących prywatności i ochrony danych. Takie środki mogą obejmować (bez ograniczeń) przekazywanie Pani/Pana danych osobowych odbiorcy w kraju, który zgodnie z decyzją Komisji Europejskiej zapewnia odpowiednią ochronę danych osobowych. W przypadku gdy przepisy dotyczące ochrony danych osobowych w innych krajach nie są tak rygorystyczne jak w tym kraju, w celu zastosowania się do przetwarzania danych osobowych przez stronę będącą adresatem w krajach spoza EOG, które nie zapewniają odpowiedniego poziomu ochrony danych, zostaną podpisane odpowiednie standardowe klauzule wymagane w UE. Pani/Pana dane osobowe będą przechowywane w bazach danych do końca eksperymentu i przez okres wymagany prawem. Sponsor i jego przedstawiciele podejmą wszelkie uzasadnione kroki w celu ochrony Pani/Pana prywatności zgodnie z wymogami prawa, w tym powiadomią Panią/Pana, jakie zabezpieczenia są podejmowane w celu zapewnienia bezpieczeństwa Pani/Pana danych osobowych. Niektóre sposoby ochrony danych osobowych obejmują wprowadzenie przez ośrodek badań klinicznych odpowiednich ustaleń dotyczących bezpieczeństwa danych osobowych, usunięcie identyfikatorów danych osobowych lub ich szyfrowanie lub kodowanie kluczem, aby nie można było ich zidentyfikować, i gromadzenie wyłącznie niezbędnych danych osobowych. Może Pani/Pan poprosić o informacje na temat tych zabezpieczeń za pośrednictwem lekarza prowadzącego eksperyment.

Powinna/nien Pani/Pan mieć świadomość, że niektóre kraje mogą nie oferować takiego samego poziomu ochrony danych osobowych jak kraj, w którym Pani/Pan mieszka lub w którym prowadzone jest eksperyment. Jednak Ośrodek kliniczny będzie postępować z danymi osobowymi, które otrzyma zgodnie ze standardem poufności dopuszczalnym według stosownego prawa polskiego i RODO. Firma PRATIA S.A. MTZ Clinical Research powered by Pratia podpisała też umowy z trzecimi stronami pracującymi dla firmy w celu zapewnienia właściwej ochrony Pani/Pana danych oraz próbek.

Informacja dla Uczestnika i Formularz Świadomej Zgody są podpisywane przez Panią/Pana w dwóch jednobrzmiących egzemplarzach, z których jeden pozostaje w PRATIA S.A., MTZ Clinical Research powered by Pratia i zostaje dołączony do akt eksperymentu, zaś drugi egzemplarz zostaje przekazany Pani/Panu.

Zgodnie z obowiązującym prawem w Polsce, podmiot opieki zdrowotnej jest obowiązany przekazać odpowiedniemu wojewódzkiemu oddziałowi Narodowego Funduszu Zdrowa informację o numerze PESEL uczestnika eksperymentu, a w przypadku jego braku - numerze dokumentu potwierdzającego jego tożsamość. W związku z tym, PRATIA S.A., MTZ Clinical Research powered by Pratia poinformuje odpowiedni krajowy oddział NFZ o Pani/Pana udziale w eksperymencie, podając Pani/Pana numer PESEL w ciągu 14 dni od daty włączenia do eksperymentu.

**Publiczna dostępność informacji z eksperymentu medycznego**

Upublicznienie informacji o tym eksperymencie zależy od decyzji sponsora, tj. firmy ECLAT srl.

**FORMULARZ ŚWIADOMEJ ZGODY**

| **Badanie wskaźnika zastąpienia konwencjonalnych papierosów przez e-papierosy i jego wpływ na biomarkery narażenia oraz ocena potencjalnych szkód zdrowotnych u użytkowników obu rodzajów papierosów**  **Kod eksperymentu medycznego: P-0101** |
| --- |

**Numer kwalifikacyjny Uczestnika: |____|____|____|____|**

Imię i nazwisko Uczestnika (**WIELKIMI LITERAMI, wypełnia Uczestnik**)

......................……………………………………………………………………………………

Potwierdzam, że:

- Lekarz-Badacz
  (**pełne imię i nazwisko Badacza, wpisuje WIELKIMI LITERAMI Uczestnik lub Badacz**)
  wyczerpująco poinformował/a mnie o celach, rodzaju, charakterze eksperymentu medycznego oraz o ryzyku i korzyściach związanych z udziałem w tym eksperymencie medycznym.
- Otrzymałam/otrzymałem jeden egzemplarz „Informacji dla Uczestnika i Formularza Świadomej Zgody”, wersja 1.0 z dnia 22.01.2024 r, oraz zapoznałem/zapoznałam się ze wszystkimi zawartymi w tym dokumencie informacjami.
- Miałam/miałem możliwość zapoznania się z warunkami ubezpieczenia odpowiedzialności cywilnej Ośrodka Badań Klinicznych przeprowadzającego eksperyment medyczny zgodnymi z polisą numer **908211598187** wystawioną przez Towarzystwo Ubezpieczeń i Reasekuracji WARTA S.A. rondo I. Daszyńskiego 1, 00-843 Warszawa, i je akceptuję.
- Miałam/ miałem możliwość zadania pytań dotyczących udziału w eksperymencie i na wszystkie pytania uzyskałem/uzyskałam zadawalające odpowiedzi.
- Rozumiem, że w każdej chwili mogę zrezygnować z udziału w eksperymencie, nawet bez podania przyczyny.
- Zostałem poinformowana/poinformowany, że eksperyment odbędzie się w Ośrodku PRATIA S.A.,MTZ Clinical Research powered by Pratia w Warszawie, ul. Gładka 22 w Warszawie, pod ścisłym nadzorem medycznym.
- Zgadzam się na ścisłe przestrzeganie Regulaminu Ośrodka Klinicznego MTZ Clinical Research, wszystkich poleceń Lekarza-Badacza i personelu Ośrodka oraz wymogów eksperymentu, w którym będę uczestniczył/a.
- Zgadzam się na sprawdzenie mojego stanu zdrowia oraz pobrania krwi i moczu w celu zakwalifikowania do eksperymentu oraz w jego trakcie i oświadczam, że wszystkie podane przeze mnie dane dotyczące mojego stanu zdrowia są prawdziwe.
- Zgadzam się na pobranie w trakcie całego eksperymentu około 40 ml krwi oraz dodatkowych próbek krwi w razie konieczności powtórzenia badań laboratoryjnych (na wizycie kwalifikacyjnej i/lub kończącej eksperyment).
- Nie przyjmuję narkotyków (substancji psychoaktywnych) i nie nadużywam alkoholu.
- W sytuacji zagrażającej mojemu zdrowiu lub życiu wyrażam zgodę na udzielenie mi niezbędnej, fachowej pomocy medycznej.
- Wyrażam zgodę na gratyfikację finansową za uczestnictwo w eksperymencie w wysokości 2 100 PLN brutto (grupa A) lub 1 000 PLN brutto (grupa B) za ukończenie eksperymentu, zgodnie z informacją zawartą w „Informacji dla Uczestnika”.
- W przypadku, gdy mój udział w eksperymencie zostanie przerwany z powodu problemów zdrowotnych po zastosowaniu badanego produktu, wyrażam zgodę na otrzymanie gratyfikacji finansowej proporcjonalnej do mojego wkładu w eksperymencie - 250 zł brutto za dzień wizyty.
- Wyrażam zgodę na gratyfikację finansową w wysokości 200 PLN brutto jeżeli spełnię wszystkie kryteria włączenia i nie spełnię żadnego z kryteriów wyłączenia w dniu 0 i będę dostępny aż do końca wizyty w tym dniu, ale nie zostanę włączony do eksperymentu, zgodnie z informacją zawartą w Informacji dla Uczestnika.
- Wyrażam zgodę na gratyfikację finansową za uczestnictwo w eksperymencie w wysokości 100 PLN brutto w przypadku zrezygnowania z dalszego udziału w eksperymencie .
- Zgadzam się na przekazanie Narodowemu Funduszowi Zdrowia mojego numeru PESEL oraz informacji o uczestnictwie w eksperymencie.
- Podpisując niniejszy dokument, nie tracę żadnych praw przysługujących mi na mocy polskiego prawa.
- Znam przysługujące mi prawa oraz moje zobowiązania wynikające z uczestnictwa w eksperymencie.

Wyrażam zgodę |__| **TAK**; |__| **NIE.**

Na moje życzenie informacja o moim uczestnictwie w eksperymencie będzie przekazana do mojego lekarza pierwszego kontaktu.

Wyrażam zgodę |__| **TAK**; |__| **NIE.**

**_____________________________________________________________________________**

**Dotyczy wyłącznie kobiet w wieku rozrodczym**

- Wyrażam zgodę na przeprowadzanie testów w przypadku gdybym w trakcie mojego udziału w eksperymencie zaszła w ciążę.

Wyrażam zgodę |__| **TAK**; |__| **NIE.**

- Wyrażam zgodę na udzielenie informacji na temat mojej ciąży, porodu i nowo narodzonego dziecka (do 45 dni po urodzeniu, w przypadku normalnego porodu, lub przez dłuższy okres w przypadku wystąpienia nieprawidłowości).

Wyrażam zgodę |__| **TAK**; |__| **NIE.**

- Zgadzam się, żeby mój lekarz mógł ujawnić zespołowi badawczemu wszelkie istotne informacje będące w jego posiadaniu, a dotyczące mojego stanu zdrowia, ciąży i mojego nienarodzonego dziecka. W zaistniałej sytuacji podam dane kontaktowe do mojego lekarza.

Wyrażam zgodę |__| **TAK**; |__| **NIE.**

**Rozumiem, że moja zgoda na dostarczenie informacji o mojej ciąży i jej wyniku jest dobrowolna. W przypadku odmowy wyrażenia zgody na zbieranie informacji o mojej ciąży i jej wyniku nadal będę uprawniona do udziału w eksperymencie medycznym.**

**_________________________________________________________________________________**

**Niniejszym potwierdzam świadomą i dobrowolną zgodę na udział w wymienionym eksperymencie medycznym. Jednocześnie wiem, że w każdej chwili mogę wycofać się z dalszego udziału w eksperymencie medycznym bez podania przyczyny. Rozumiem i akceptuję informacje i zobowiązania zawarte w niniejszej „Informacji dla Uczestnika i Formularzu Świadomej Zgody” związane z uczestniczeniem w eksperymencie medycznym P-0101.**

**Wypełnia Uczestnik: _____________________________________________________________**

Pełne imię i nazwisko (WIELKIMI LITERAMI, wypełnia Uczestnik badania)

**Warszawa** |__|__| |__|__| |__|__|__|__| |__|__|:|__|__|

Miejsce dd mm rrrr godz. min. Podpis Uczestnika

**Oświadczenie badacza/współbadacza uzyskującego zgodę:**

- Dokładnie omówiłam/omówiłem z uczestnikiem albo przeczytałem/przeczytałam uczestnikowi treść informacji. Zgodnie z moją najlepszą wiedzą upewniłem/upewniłam się, że osoba ta dobrze rozumie charakter tego eksperymentu medycznego oraz zagrożenia i korzyści związane z udziałem w eksperymencie medycznym.
- Potwierdzam, że uczestnik miał dość czasu na zadawanie pytań dotyczących eksperymentu medycznego i że na wszystkie te pytania odpowiedziałem/odpowiedziałam poprawnie i zgodnie z moją najlepszą wiedzą. Potwierdzam, że uczestnik nie został zmuszony do wyrażenia zgody, a zgoda została wyrażona w sposób swobodny i dobrowolny.
- Potwierdzam, że przekazano uczestnikowi podpisany oryginał niniejszej informacji i formularza zgody.

**Wypełnia Lekarz-Badacz___________________________________________________________**

Pełne imię i nazwisko (WIELIMI LITERAMI, wypełnia Lekarz-Badacz)

**Warszawa** |__|__| |__|__| |__|__|__|__| |__|__|:|__|__|

Miejsce dd mm rrrr godz. min. Podpis Lekarza-Badacza

**ZGODA NA PRZETWARZANIE DANYCH OSOBOWYCH I MEDYCZNYCH**

**UCZESTNIKA EKSPERYMENTU MEDYCZNEGO**

| **Badanie wskaźnika zastąpienia konwencjonalnych papierosów przez e-papierosy i jego wpływ na biomarkery narażenia oraz ocena potencjalnych szkód zdrowotnych u użytkowników obu rodzajów papierosów**  **Kod eksperymentu medycznego: P-0101** |
| --- |

Ja, niżej podpisany,

………………………………………………………………

(imię i nazwisko należy napisać WILEKIMI LIETARMI)

**Niniejszym wyrażam zgodę** ❑

na przetwarzanie moich danych osobowych w postaci:

- informacji, które bezpośrednio mnie identyfikują, takie jak imię i nazwisko, PESEL i wiek,
- informacji, które pozwalają na kontakt ze mną, takie jak numery telefonów, adres/y e-mail, adres zamieszkania/adres do korespondencji, imię i nazwisko oraz numer telefonu i adres osoby wskazanej przeze mnie do kontaktu,
- informacji, które pozwalają na przekazanie mi płatności za eksperyment medyczny, takie jak numer rachunku bankowego,
- danych szczególnej kategorii w zakresie określonym protokołem eksperymentu medycznego,

które są niezbędne w celu prowadzenia eksperymentu medycznego o numerze Protokołu Sponsora P-0101 przez ECLAT srl z siedzibą główną w Via S. Sofia 89, 95123 Catania, Włochy.

Zgadzam się, by moje dane osobowe były zbierane i procesowane jedynie przez osoby uprawnione przez Sponsora.

Zgadzam się, by moje dane osobowe, z wyłączeniem danych medycznych, były zbierane i przetwarzane również do celów realizacji płatności związanej z gratyfikacją finansową za udział w niniejszym eksperymencie medycznym.

Zgadzam się, by moje zakodowane dane, zarówno w formie informacji, jak i próbki krwi, pochodzące z tego eksperymentu medycznego były przekazywane do krajów w których obowiązujące zasady ochrony danych osobowych nie są takie same jak te obowiązujące w Polsce.

Zgadzam się na dostęp Lekarzy-Badaczy do mojej dokumentacji medycznej zawierającej dane osobowe i medyczne, także obejmujące okres przed rozpoczęciem udziału w eksperymencie medycznym.

**Warszawa** |__|__| |__|__| |__|__|__|__| |__|__|:|__|__|

Miejsce dd mm rrrr godz. min. Podpis Uczestnika

**INFORMACJE DOTYCZĄCE PRZETWARZANIA DANYCH OSOBOWYCH**

**UCZESTNIKA EKSPERYMENTU MEDYCZNEGO**

Zgodnie z artykułem 13 ust. 1 oraz ust. 2 Rozporządzenia Parlamentu Europejskiego i Rady (UE) 2016/679 z dnia 27 kwietnia 2016 r. w sprawie ochrony osób fizycznych w związku z przetwarzaniem danych osobowych i w sprawie swobodnego przepływu takich danych oraz uchylenia dyrektywy 95/46/WE (ogólne rozporządzenie o ochronie danych z dnia 27 kwietnia 2016 r., dalej: „RODO“), niniejszym informujemy, że:

1. Administratorem Pani/Pana danych osobowych, tj.

- informacji, które bezpośrednio Panią/Pana identyfikują, takie jak imię i nazwisko, PESEL i wiek,
- informacji, które pozwalają na kontakt z Panią/Panem, takie jak numery telefonów, adres/y e-mail, adres zamieszkania/adres do korespondencji, imię i nazwisko oraz numer telefonu i adres osoby wskazanej przez Panią/Pana do kontaktu,
- informacji, które pozwalają na przekazanie Pani/Panu płatności za eksperyment medyczny,
- danych szczególnej kategorii w zakresie określonym protokołem eksperymentu medycznego, przetwarzanych w związku z Pani/Pana włączeniem i udziałem w eksperymencie medycznym P-0101 (dalej nazywanym „Eksperymentem”),

jak również odpowiedzialny za archiwizację Pani/Pana danych jest Sponsor: ECLAT srl
z siedzibą główną w Via S. Sofia 89, 95123 Catania, Włochy.

1. Pani/Pana dane osobowe będą przetwarzane do celów prowadzenia eksperymentu, zgodnie z wyrażoną przez Panią/Pana zgodą, stosowanie do treści artykułu 9, ust. 2, litera (a) RODO
2. W przypadku jakichkolwiek zapytań, wątpliwości, wniosków lub żądań dotyczących przetwarzania Pani/Pana danych przez Sponsora, może Pani/Pan skontaktować się z osobą odpowiedzialną za ochronę danych osobowych w PRATIA S.A., MTZ Clinical Research powered by Pratia, ośrodku który jest podmiotem przetwarzającym Pana/Pani dane osobowe, wysyłając wiadomość na adres [privacy@pratia.com](mailto:privacy@pratia.com).
3. Odbiorcami Pani/Pana danych osobowych mogą być:
4. PRATIA S.A., MTZ Clinical Research powered by Pratia., z siedzibą w Warszawie 02-172, ul. Gładka 22, podmiot medyczny zarejestrowany w PRATIA S.A. KRS: 0000428924, NIP: 1182086113, jako ośrodek badawczy, który prowadzi eksperyment na zlecenie administratora danych, jego upoważnieni pracownicy, współpracownicy, partnerzy technologiczni, logistyczni i organizacyjni oraz lekarze prowadzący eksperyment;
5. Sponsor eksperymentu i monitorzy działający na zlecenie Sponsora, CRO (organizacji realizującej na zlecenie Sponsora określone zadania związane z eksperymentem), przedstawiciele, osoby fizyczne i prawne, będące współpracownikami lub partnerami Sponsora (np. osobom wynajętym przez sponsora do przeprowadzenia audytu);
6. Właściwa komisja bioetyczna (organ odpowiedzialny za ochronę Pani/Pana praw i bezpieczeństwa jako uczestnika eksperymentu);
7. Urzędy państwowe odpowiedzialne za bezpieczeństwo uczestników eksperymentu
8. Odpowiednie władze mogące sprawdzić dane identyfikujące Panią/Pana.
9. Pani/Pana pseudonimizowane dane osobowe zebrane w trakcie eksperymentu mogą być przesyłane poza UE i EOG (do kraju trzeciego), jeśli zgodnie z decyzją Komisji Europejskiej taki kraj trzeci, terytorium, sektor lub organizacja w kraju trzecim zapewnia odpowiedni poziom ochrony danych. Jeśli Komisja Europejska nie wyda takiej decyzji, administrator danych może przesłać Pani/Pana dane do kraju trzeciego tylko, jeśli administrator danych lub podmiot przetwarzający dane zapewni odpowiednią ochronę danych i pod warunkiem, że w takim kraju obowiązują odpowiednie prawa i możliwość ich wyegzekwowania przez Panią/Pana. Jeśli Komisja Europejska nie wyda takiej decyzji i nie obowiązują zabezpieczenia wspomniane w poprzednim zdaniu, Pani/Pana dane mogą być przesyłane wyłącznie na podstawie Pani/Pana jednoznacznej zgody, pod warunkiem spełnienia wymagań określonych w artykule 49 ust. 1 i ust. 2 RODO.
10. Pani/Pana dane osobowe będą przechowywane przez okres czasu niezbędny do przeprowadzenia i dokumentowania eksperymentu, w tym przez okres czasu zgodny z obowiązującymi przepisami prawnymi. Natomiast podstawowa dokumentacja eksperymentu nie będzie przechowywana dłużej niż przez 25 lat, licząc od początku roku kalendarzowego następującego po roku, w którym zakończono eksperyment.
11. W celu skorzystania z przysługujących Pani/Panu praw może Pani/Pan w każdym momencie zgłosić do administratora danych albo do ośrodka badań klinicznych (podmiot przetwarzający dane, który przekaże Państwa wniosek do administratora danych) poprzez [privacy@pratia.com](mailto:privacy@pratia.com) wniosek o przedstawienie informacji i wyjaśnień dotyczących przetwarzania danych osobowych;
12. Ma Pani/Pan prawo dostępu do podanych przez Panią/Pana danych osobowych, ich sprostowania, usunięcia lub ograniczenia ich przetwarzania, oraz prawo do wniesienia sprzeciwu wobec ich przetwarzania, a także prawo do ich przenoszenia.
13. Ma Pani/Pan prawo do wycofania swojej zgody w dowolnym momencie, w wyniku czego nie poniesie Pani/Pan żadnej kary ani nie straci przysługujących Pani/Panu uprawnień; zgodę można wycofać w dowolnej postaci, tj. e-mailem, na piśmie, przez telefon. Wycofanie zgody nie będzie miało wpływu na zgodność z prawem przetwarzania danych przed jej wycofaniem.
14. Ma Pani/Pan prawo zgłosić skargę do organu nadzorczego (Prezesa Urzędu Ochrony Danych Osobowych), jeśli uzna Pani/Pan, że Pani/Pana dane osobowe są przetwarzane z naruszeniem obowiązujących przepisów.
15. Na podstawie podanych przez Panią/Pana danych osobowych nie będą podejmowane żadne automatyczne decyzje, w tym profilowanie.
16. Podanie przez Panią/Pana danych osobowych jest dobrowolne, ale niezbędne do wzięcia udziału w eksperymencie. Brak Pani/Pana zgody na przetwarzanie Pani/Pana danych osobowych będzie skutkowało brakiem możliwości wzięcia przez Panią/Pana udziału w eksperymencie.

**Warszawa** |__|__| |__|__| |__|__|__|__| |__|__|:|__|__|

Miejsce dd mm rrrr godz. min. Podpis Uczestnika
