# Supplementary material for: Statistical analyses plan for “MAGnItude of cigarette substitutioN after initiation oF e-cigarettes and its ImpaCt on biomArkers of exposure and potenTial harm in dual users”: MAGNIFICAT trial
Source: Heliyon. 2024 Oct 22;10(21):e39695. doi: 10.1016/j.heliyon.2024.e39695 (PMC11565015; doi:10.1016/j.heliyon.2024.e39695)
Supplement: Multimedia component 2 [file mmc2.pdf]

Komisja Bioetyczna

Okręgowej Izby Lekarskiej w Warszawie

Uchwała Nr 04/24

Komisji Bioetycznej

Okręgowej Izby Lekarskiej w Warszawie

z dnia 22 lutego 2024

w sprawie wydania opinii o projekcie eksperymentu medycznego o nr rejestru KB/1481/24

Na podstawie art. 29 ust. 1 zdanie 1 ustawy z dnia 5 grudnia 1996 r. o zawodzie lekarza i lekarza dentysty (t.j. Dz. U. z 2023 r., poz. 1516 z późn. zm.):

§ 1

1. Komisja Bioetyczna przy Okręgowej Izbie Lekarskiej w Warszawie w składzie:
   1) przewodniczący – prof. dr hab. n med. Stanisław Niemczyk
   2) z-ca – praw. Sebastian Stykowski
   3) sekretarz – dr n. med. Barbara Dobies – Krześniak
   4) dr hab. n med. Paweł Balsam
   5) ks. prof. dr hab. Dariusz Pater
   6) lek. Marek Pawliszak
   7) mgr Renata Piasecka - Krawczyk
   8) dr n med. Joanna Romejko - Jarosińska
   9) dr n med. Michał Stępka
   10) dr n. med. Marek Stopiński

- na posiedzeniu w dniu 22 lutego 2024 r. **wydaje pozytywną opinię o projekcie eksperymentu medycznego** pod tytułem *„Badanie wskaźnika zastąpienia konwencjonalnych papierosów przez e-papierosy i jego wpływ na biomarkery narażenia oraz ocena potencjalnych szkód zdrowotnych u użytkowników obu rodzajów papierosów”*. nr protokołu P-0101, koordynowanego przez lek. Tomasz Kłosa, które będzie prowadzone w MTZ Clinical Research powered by Pratia, Pratia S.A. ul. Gładka 22, 02-172 Warszawa pod **warunkiem: zmiany kryterium:**

Zamiast:

Kobiety w ciąży, kobiety karmiące piersią lub kobiety chcące zajść w ciążę w trakcie eksperymentu (zostanie wykonany test ciążowy z próbki moczu)

Proponujemy:

Kobiety w ciąży, kobiety karmiące piersią lub kobiety w wieku rozrodczym nie stosujące skutecznej antykoncepcji (zostanie wykonany test ciążowy z próbki moczu).

2. MTZ Clinical Research powered by Pratia, Pratia S.A. ul. Gładka 22, 02-172 Warszawa NIP: 118-208-61-13, zobligowany/zobligowana jest do utrzymania ważnej polisy ubezpieczeniowej OC lub innego dokumentu ubezpieczenia przez cały okres prowadzenia badania.

§2

Uchwała wchodzi w życie z dniem podjęcia.

UZASADNIENIE:

Komisja Bioetyczna stwierdza, że złożony wniosek zawiera kompletną dokumentację, w tym w szczególności:

Sekcja 1:

List przewodni z dnia 06.02.2024 r.

Sekcja 2:

Wniosek do Komisji Bioetycznej o wydanie opinii o projekcie eksperymentu medycznego z dnia 06.02.2024 r. (we wniosku znajdują się elektroniczne podpisy z datami w formacie mm.dd.rrrr)

Oświadczenie z dnia 22.01.2024 r. dotyczące zasad rekrutacji Uczestników eksperymentu medycznego podpisane przez Głównego Badacza.

Pakiet ogłoszeń do badania P-0101 (wersja 1.0 z dnia 19.01.2024 r.), który obejmuje wzory ogłoszeń:

- Ogłoszenie na strony internetowe
- Ogłoszenie na plakat/ulotkę/ogłoszenie w gazecie /media społecznościowe
- Informacja o badaniu- e-mail/sms
- Informacja o wizycie kwalifikacyjnej - e-mail/sms
- Informacja o zakwalifikowaniu się do badania - e-mail/sms
- Przypomnienie o rozpoczęciu badania/przypomnienie odnośnie wizyt ambulatoryjnych - e-mail/sms

Sekcja 3:

Podpisany i opatrzony datą 17.01.2024 r. życiorys Głównego Badacza w języku polskim, oryginał wraz z opisem jego działalności zawodowej i naukowej. Dołączono również prawo wykonywania zawodu z informacją o ukończeniu specjalizacji, certyfikat ze szkolenia GCP oraz dyplom doktorski.

Sekcja 4:

Informacja dla Uczestnika i Formularz Świadomej Zgody, Wersja 1.0 z dnia 22.01.2024 r. zawierający:

- informację o gratyfikacjach dla uczestników badania
- informację na temat ubezpieczenia odpowiedzialności cywilnej podmiotu przeprowadzającego eksperyment medyczny
- wzór zgody na przetwarzanie danych osobowych i medycznych Uczestnika eksperymentu medycznego

Dodatkowo zostały dołączone następując dokumenty:

1) Karta Uczestnika o numerze protokołu P-0101, wersja 1.0 z dnia 22.01.2024 r.
2) Regulamin obowiązujący Uczestników Badań w Ośrodku Badań Klinicznych MTZ Clinical Research powered by Pratia z dnia 29.07.2022 r.
3) Formularze dla pacjentów:

- Test motywacji do rzucenia palenia (MTSS), wersja 1.0 z dnia 22.01.2024 r.
- Kwestionariusz intencji użycia i zadowolenia z produktu badanego (BIIC), wersja 1.0 z dnia 22.01.2024 r.
- Skala postrzeganego ryzyka zdrowotnego (PRI-P), wersja 1.0 z dnia 22.01.2024 r.
- Test uzależnienia od papierosów wg Fagerströma (FTCD), wersja 1.0 z dnia 22.01.2024 r.
- Skala oceny nasilenia dolegliwości ze strony układu oddechowego (RSES), wersja 1.0 z dnia 22.01.2024 r.

Sekcja 5:

Specyfikacja produktu badanego

Sekcja 6:

Instrukcja użytkowania aplikacji do zbierania informacji na temat ilości wypalonych papierosów tradycyjnych i/lub e-papierosów na potrzeby realizacji projektu „MAGNIFICAT", wersja 1.0 z dnia 15.01.2024 r.

Sekcja 7:

Projekt karty obserwacji klinicznej, wersja 1.0 z dnia 05.02.2024 r.

Sekcja 8:

Wzór etykiet produktu wykorzystanego do badania P-0101, wersja 1.0 z dnia 05.02.2024 r.

Sekcja 9:

Kopia polisy obowiązkowego ubezpieczenia odpowiedzialności cywilnej podmiotu przeprowadzającego eksperyment medyczny wystawiona w dniu 05.02.2024 r. przez **Towarzystwo**

Ubezpieczeń i Reasekuracji WARTA S.A. rondo I. Daszyńskiego 1, 00-843 Warszawa numer polisy: 908211598187 wraz z warunkami ubezpieczenia.
Kopie potwierdzenia opłacenia składki ubezpieczeniowej w dniach 06.02.2024 r oraz 07.02.2024 .

Sekcja 10:
Streszczenie protokołu eksperymentu medycznego w języku polskim, wersja 1.0 z dnia 19.01.2024 r.

Sekcja 11:
Protokół eksperymentu medycznego, wersja 1.0 z dnia 19.01.2024 r. wraz ze stronami podpisów przedstawiciela Sponsora oraz Głównego Badacza.

Sekcja 12:
Informacja o Ośrodku Badawczym z dnia 17.01.2024 r. dotycząca kwalifikacji personelu, który będzie uczestniczył w prowadzeniu eksperymentu wraz z informacją na temat wyposażenia Ośrodka podpisana przez Głównego Badacza.
Załączono oryginały życiorysów badaczy uczestniczących w eksperymencie. Dołączono również dyplomy, prawa wykonywania zawodu (z informacją o ukończeniu specjalizacji jeśli dotyczy) i certyfikaty ze szkolenie GCP.

Sekcja 13:
Zgoda Kierownika Ośrodka Badań Klinicznych na prowadzenie eksperymentu medycznego z dnia 22.01.2024 r.

Sekcja 14:
Umowa o przeprowadzenie eksperymentu medycznego między firmami ECLAT srl i PRATIA S.A. obowiązująca od 28.11.2023 r. podpisana przez przedstawicieli obu stron.

Ocena zasadności medycznej projektu eksperymentu została przygotowana przez specjalistę onkologa klinicznego. Recenzent po szczegółowej analizie dokumentacji badania stwierdził, że przedstawione założenia projektu badawczego i jego realizacja są zasadnie. Plan badania przedstawiony jest w sposób rzetelny i zgodny z przyjętymi zasadami, standardami
i kryteriami. Członkowie komisji byli zgodni w tym, że badanie ma podstawy merytoryczne i zasługuje na prowadzenie pod warunkiem wprowadzenia zmiany w kryterium wykluczenia z badania z sformułowania „Kobiety w ciąży, kobiety karmiące piersią lub kobiety chcące zajść w ciążę w trakcie eksperymentu (zostanie wykonany test ciążowy z próbki moczu)”
na:

„Kobiety w ciąży, kobiety karmiące piersią lub kobiety w wieku rozrodczym nie stosujące skutecznej antykoncepcji (zostanie wykonany test ciążowy z próbki moczu)”.

(W/w dokumenty zostały wyszczególnione według informacji zawartych we wniosku złożonym przez głównego badacza)

Komisja Bioetyczna przypomina, że obowiązkiem głównego badacza jest :

1. zgłoszenia wszelkich zmian i odchyleń w protokole eksperymentu medycznego,
2. zgłoszenia wszelkich nowych informacji wiążących się z niekorzystnym wpływem na bezpieczeństwo osób biorących udział w eksperymencie oraz na jego przebieg,
3. zgłoszenia wszelkich ciężkich lub nieoczekiwanych niepożądanych działań leków (ADR) a także ciężkich zdarzeń niepożądanych (SAE)
4. informowania o decyzjach innych komisji bioetycznych,
5. sporządzania rocznych raportów z przebiegu eksperymentu (nie później niż do końca grudnia każdego roku)
6. informowania o zakończeniu eksperymentu i jego wynikach, w tym wymóg dostarczenia kopii ostatecznej wersji raportu z eksperymentu po jego zakończeniu.

Wszelka korespondencja musi być przekazywana na piśmie listem poleconym bądź za potwierdzeniem odbioru.

Tekst uchwały został sporządzony w 2 jednobrzmiących egz. po jednym dla wnioskodawcy i Komisji Bioetycznej.

Skład i działanie Komisji Bioetycznej jest zgodne z Wskazówkami i Zaleceniami dla Europejskich Komisji Etycznych opracowanymi przez EFGCP, Zasadami Prawidłowego Prowadzenia Badań Klinicznych (GCP) oraz wymogami lokalnymi.

Zastępca Przewodniczącego
Komisji Bioetycznej
przy Okręgowej Izbie Lekarskiej w Warszawie

....
dr n. pr. Sebastian Stykowski
Podpis Przewodniczącego

Pouczenie:

Zgodnie z art. 29 ust. ustawy z dnia 5 grudnia 1996 r. o zawodach lekarza i lekarza dentysty (t.j. Dz. U. z 2023 r., poz. 1516z późn. zm.) odwołanie od uchwały komisji bioetycznej wyrażającej opinię może wnieść:

1) wnioskodawca;
2) kierownik podmiotu, w którym eksperyment medyczny ma być przeprowadzony;

3) komisja bioetyczna właściwa dla ośrodka, który ma uczestniczyć w wieloośrodkowym eksperymencie medycznym.

Odwołanie wnosi się za pośrednictwem komisji bioetycznej, która podjęła uchwałę, do Odwoławczej Komisji Bioetycznej ul. Miodowa 15, 00-952 Warszawa w terminie 14 dni od dnia otrzymania uchwały wyrażającej opinię.
